# Recent Advances on the Halo- and Cyano-Trifluoromethylation of Alkenes and Alkynes

**DOI:** 10.3390/molecules26237221

**Published:** 2021-11-28

**Authors:** Bo Fu, Jorge Escorihuela, Jianlin Han, Santos Fustero, Pablo Barrio, Mikiko Sodeoka, Shintaro Kawamura, Alexander Sorochinsky, Vadim A. Soloshonok

**Affiliations:** 1 Jiangsu Co-Innovation Center of Efficient Processing and Utilization of Forest Resources, College of Chemical Engineering, Nanjing Forestry University, Nanjing 210037, China; fubo@njfu.edu.cn; 2 Departamento de Química Orgánica, Universidad de Valencia, 46100 Valencia, Spain; 3 Departamento de Química Orgánica e Inorgánica, Universidad de Oviedo, 33006 Oviedo, Spain; barriopablo@uniovi.es; 4 RIKEN Center for Sustainable Resource Science, Catalysis and Integrated Research Group, and RIKEN Cluster for Pioneering Research, Synthetic Organic Chemistry Laboratory, Saitama 351-0198, Japan; sodeoka@riken.jp (M.S.); skawamura@riken.jp (S.K.); 5 V.P. Kukhar Institute of Bioorganic Chemistry and Petrochemistry, The National Academy of Sciences of Ukraine, 02094 Kyiv, Ukraine; sorochinsky.a@gmail.com; 6 Department of Organic Chemistry I, Faculty of Chemistry, University of the Basque Country UPV/EHU, 20018 San Sebastián, Spain; vadym.soloshonok@ehu.eus; 7 IKERBASQUE-Basque Foundation for Science, Alameda Urquijo 36-5, Plaza Bizkaia, 48011 Bilbao, Spain

**Keywords:** difunctionalization, cyanotrifluoromethylation, halotrifluoromethylation, alkenes, alkynes, synthetic methods

## Abstract

Incorporation of fluorine into organic molecules is a well-established strategy in the design of advanced materials, agrochemicals, and pharmaceuticals. Among numerous modern synthetic approaches, functionalization of unsaturated bonds with simultaneous addition of trifluoromethyl group along with other substituents is currently one of the most attractive methods undergoing wide-ranging development. In this review article, we discuss the most significant contributions made in this area during the last decade (2012−2021). The reactions reviewed in this work include chloro-, bromo-, iodo-, fluoro- and cyano-trifluoromethylation of alkenes and alkynes.

## 1. Introduction

**Figure 1:**
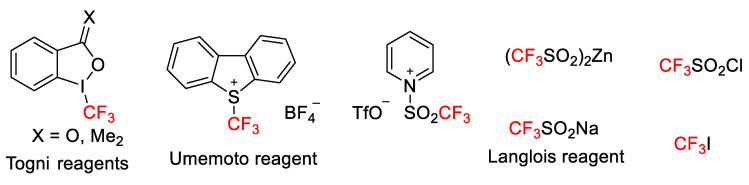
Trifluoromethylating reagents.

The incorporation of fluorine atoms or fluorinated groups into appropriate positions in pharmaceuticals has been established as a challenging strategy in medicinal chemistry and several fluorinated-containing pharmaceuticals, such as Celecoxib, Efavirenz, Odanacatib, Sitagliptin, Trifluridine, and Prozac among others, have been developed [1,2,3,4,5,6,7,8,9,10,11,12,13]. In particular, the trifluoromethyl group (-CF_3_) is a unique structural motif, which plays a crucial role in pharmaceuticals [14,15] and agrochemicals [16] due to its profound effect on properties such as absorption, distribution, metabolism, and excretion (ADME) [17]. In this sense, it is well known that the substitution of a trifluoromethyl group by a methyl group in drugs can significantly alter the chemical and biological properties of the original molecule. Therefore, the quest of simple, easy, and environmentally benign methodologies for the efficient and selective introduction of fluorine atoms or fluorinated groups into organic molecules has emerged as a highly demanded field in modern organic synthesis. Over the past decade, many processes have emerged as effective methods for the incorporation of the CF_3_-moiety into simple molecules such as non-activated olefins, alkynes, and aromatic derivatives [18,19,20,21,22,23,24]. Among these synthetic methodologies, trifluoromethylation of alkenes and alkynes represents one of the most effective routes to fluorinated compounds. Thus, it is not surprising that the development of fluorinating reagents has experienced a blossoming interest in organofluorine chemistry during the past decades. Several commercially available fluoromethylating reagents, such as Umemoto reagent [25], Togni reagent [26], Langlois reagent (one of the cheapest and most convenient reagents for trifluoromethylation), and others (Figure 1) have been reported, which usually feature high stability and reactivity.

Along with the trifluoromethyl group, the introduction of a halogen atom can give access to further versatile transformations for more complex structural manipulations in the target molecule. Conventional halotrifluoromethylation suffers from complex operations with gaseous trifluoromethylating reagents, which are generally difficult to handle, hampering the development of the process. Therefore, various alternative and more convenient strategies for halotrifluoromethylation have been explored in the past decades. In this review, we highlight the most significant contributions made along the last decade (2012–2021) involving procedures devoted mainly to the generation of halo-C/C-CF_3_ and NC-C/C-CF_3_ bonds. In this regard, we have organized the review according to the type of halogen atom introduced in the halotrifluoromethylation reaction to finally end with the different cyanotrifluoromethylation approaches.

## 2. Chloro- and Bromotrifluoromethylation

**Figure 2:**
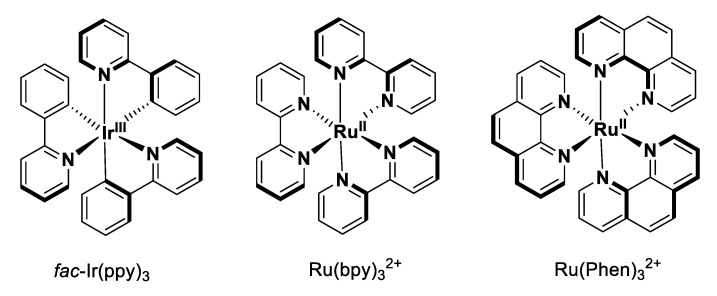
Common photocatalysts used in halotrifluoromethylation reactions.

Photoredox catalysis has undergone an exponential growth in the last two decades, probably due to the simplicity of the method, the excellent reducing ability of photocatalysis in the excited state, and in most cases, the low cost of the starting materials [27]. The use of photoredox catalysis such as the widely-used ruthenium(II) polypyridine complexes (e.g., [Ru(bpy)_3_]^2+^, bpy = 2,2′-bipyridine, or [Ru(Phen)_3_]^2+^, Phen = 1,10-phenanthroline), the well-known iridium(III) cyclometalated derivatives (e.g., *fac*-Ir(ppy)_3_, ppy = 2-phenylpyridine) has been established as an elegant and powerful tool in synthetic chemistry because they can smoothly catalyze single-electron transfer (SET) processes with visible light irradiation under operationally simple conditions at room temperature (Figure 2). Concretely, the difunctionalization of unsaturated carbon–carbon bonds using this strategy provides, among others, the formation of carbon–fluoroalkyl bonds, in particular, C–CF_3_ bonds, which are of great interest both in the agrochemical and pharmaceutical industry. The trifluoromethylation of alkenes, including halotrifluoromethylation and others, has emerged as an interesting and attractive synthetic tool for the incorporation of CF_3_ moieties in bioactive molecules [28]. Different reagents have been used to this end, with R_f_X (in particular, CF_3_I gas, b.p. – 22 °C), Togni reagent, Umemoto reagent, CF_3_SO_2_Cl (corrosive and volatile), Langlois and Baran reagents, and *N*-trifylpyridinium salts being the most widely used [29,30,31].

**Scheme 1:**
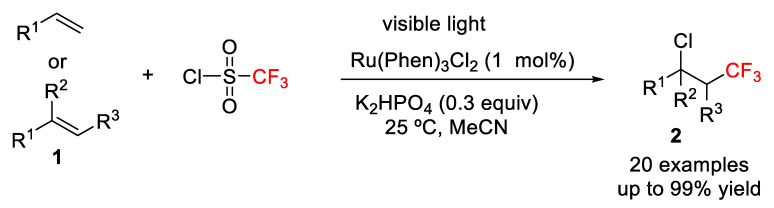
Trifluoromethyl chlorination of terminal and internal alkenes.

Quite recently, different authors used trifluoromethanesulfonyl chloride (CF_3_SO_2_Cl, b.p. 29–32 °C) as a useful, effective, and inexpensive reagent for the chlorotrifluoromethylation of terminal and internal alkenes and alkynes as it can be easily reduced to generate a trifluoromethyl radical (∙CF_3_) intermediate. In this context, in 2014, Jung, Han, and co-workers developed a mild and simple strategy for the photoredox-catalyzed vicinal difunctionalization of terminal and internal alkenes **1** using CF_3_SO_2_Cl as CF_3_ and Cl sources and releasing SO_2_ as a single byproduct (Scheme 1) [32].

The authors initially examined the difunctionalization of terminal olefins such as **1** [R^1^ = TsNH(CH_2_)_3_] using 1.5 equiv of CF_3_SO_2_Cl as CF_3_ and Cl sources under visible light irradiation. The process took place, in general, in good to excellent chemical yield during several hours in the presence of 1 mol% Ru(Phen)_3_Cl as photocatalyst (Figure 2) and K_2_HPO_4_ (0.3 equiv) as buffering reaction system. DCM and MeCN were the solvents of choice and other photocatalysts such as Ir(ppy)_3_ and Ru(bpy)_3_Cl_2_·6H_2_O also afforded good yields.

**Scheme 2:**
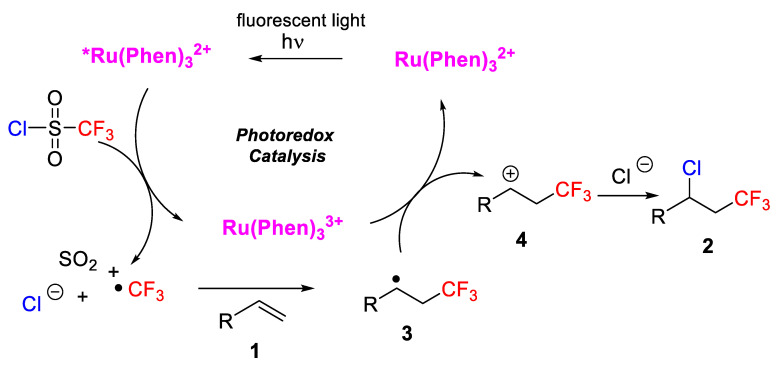
Proposed mechanism for the chlorotrifluoromethylation of alkenes.

The scope of the method was studied showing, in general, high reactivity with terminal alkenes including functionalized olefins containing groups such as *N*-Tosyl, *N*-Boc, alcohols, esters, amides, and pyridines. 1,1-Disubstituted and internal alkenes also provided high yields in the chlorotrifluoromethylation process. The authors, moreover, indicated that this protocol was also appropriate for the late-stage difunctionalization of biologically active compounds such as the pesticide rotenone and the insecticide (+)-nootkatone. A mechanism was proposed, which starts with the first irradiation of the photocatalyst Ru(Phen)_3_^2+^ with visible light to generate Ru(Phen)_3_^3+^. The CF_3_SO_2_Cl radical anion collapses to ∙CF_3_, SO_2_ and Cl^−^. Then, the CF_3_ radical was added to the alkene to form, first, a radical intermediate **3,** and then a carbocation **4**, which was finally trapped by Cl^−^ to generate the target molecule (Scheme 2).

**Scheme 3:**
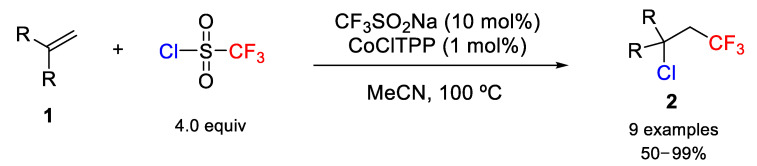
Catalytic chlorotrifluoromethylation of terminal olefins catalyzed by Co-porphyrin complexes.

More recently, Matsubara and coworkers studied the chlorotrifluoromethylation of substituted terminal olefins in the presence of highly coordinated square-planar cobalt(II) complexes, such as cobalt(II) porphyrin catalysts CoTPP (TPP= 5,10,15,20-tetraphenylporphyrinato) [33]. After checking various cobalt and copper complexes, it was found that the combination of CoClTPP and CF_3_SO_2_Na was the most efficient catalyst when the reaction was carried out in MeCN as solvent. The authors also used trifluoromethanesulfonyl chloride (CF_3_SO_2_Cl) as a CF_3_ radical source, generated after pyrolytic release of SO_2_ gas, and a chlorine source (Scheme 3). Modification of the reaction conditions showed that the combination of the Co^III^ porphyrin, easily prepared by air-oxidation of Co^II^ complexes, and Langlois’ salts (CF_3_SO_2_Na) provided good results in terms of yields, too. Under these optimized conditions, the scope of the method was studied using different terminal functionalized olefins and, in general, high chemical yields were obtained (Scheme 3).

**Scheme 4:**
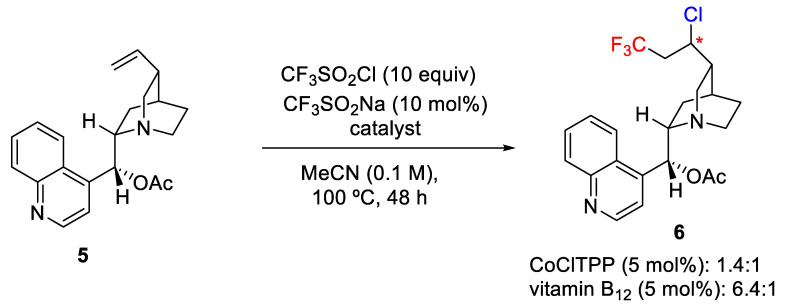
Diastereoselective chlorotrifluoromethylation of **5**.

Interestingly, the use of vitamin B_12_ (available as cyanocobalamin) was tested as Co^III^ catalyst, and it was found that the process worked quite well providing the target molecules in a reasonable yield. This procedure opens new opportunities for pharmaceutical applications and for the study of the regio- and diastereoselectivity in this kind of chlorotrifluoromethylation transformations. Thus, for example, cinchona alkaloid derivative **5** was converted into the target molecule **6** with improved chemical yield and selectivity (6.4:1) when vitamin B_12_ (5 mol%) was used as catalyst instead of the CoClTPP (5 mol%) complex (1.4:1) (Scheme 4).

**Scheme 5:**
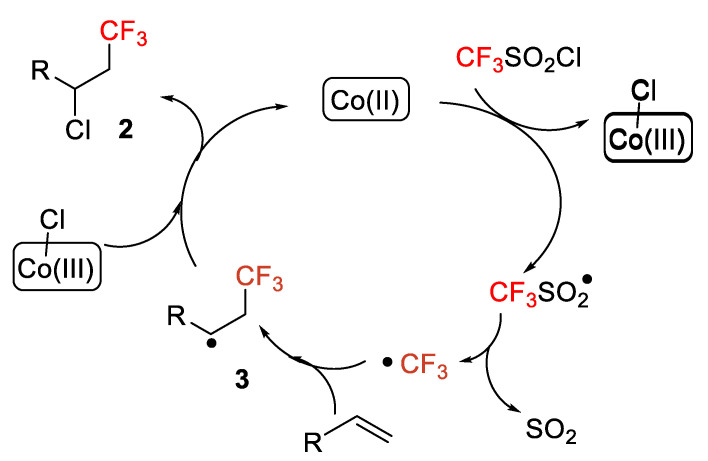
Catalytic cycle for the chlorotrifluoromethylation of olefins catalyzed by Co-complexes.

A catalytic cycle was proposed to explain the obtained results (Scheme 5).

**Scheme 6:**
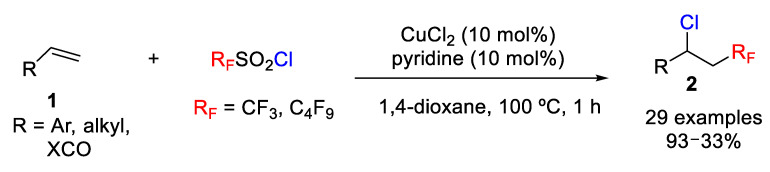
Difunctionalization of alkenes with R_F_SO_2_Cl.

Xiao, Lin et al. have recently focused on the development of simple and efficient protocols for the incorporation of various fluoroalkyl groupings, in particular a CF_3_ substituent, using simple and inexpensive catalysts consisting of a mixture of copper(II) chloride (CuCl_2_) and pyridine for the chlorotrifluoromethylation of simple aromatic alkenes, α,β-unsaturated alkenes, and aliphatic alkenes with CF_3_SO_2_Cl as CF_3_ and Cl sources [34]. Previously, Liu’s group had found that copper (I) complexes also reduced CF_3_SO_2_Cl and, under those conditions, they studied the successful aminotrifluoromethylation of alkenes. However, stoichiometric amounts of a silver salt were needed to eliminate side reactions, which limited the utility of the process [35]. In this sense, Xiao’s group discovered that copper(II) chloride could also be an efficient catalyst in this kind of process, although their first attempts resulted in low efficiency (DMF, 7% yield). They observed that the reaction conditions such as temperature, concentration, presence of a base (e.g., pyridine) and, especially, the solvent played a crucial role in achieving high yields. After testing different solvents, they found that the use of 1,4-dioxane as a solvent, high temperature (100 °C) and pyridine (10 mol%) as base provided the best reaction conditions. Other solvents, bases, copper sources as well as different temperatures were also studied but lower yields were, in general, achieved. The scope of the process was studied, and they found a high level of functional group tolerance with styrene derivatives as well as with α,β-unsaturated amides, esters, and ketones. The process also worked with alkyl olefins but resulted in lower yields. The method can be applied to chloroperfluoroalkylation transformations (Scheme 6).

**Scheme 7:**
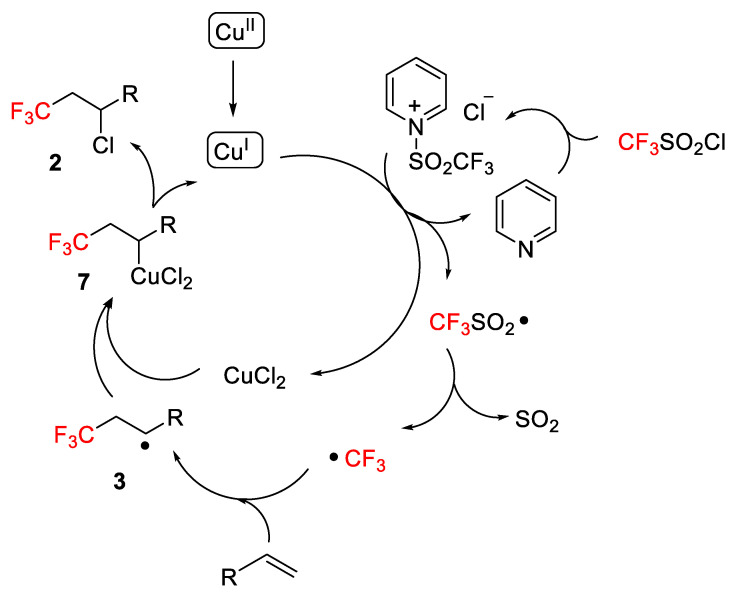
Plausible reaction mechanism catalyzed by Cu^II^.

A plausible reaction mechanism was proposed by the authors (Scheme 7). Cu^II^ was initially reduced to Cu^I^, which apparently is the real catalyst of the process. CF_3_SO_2_Cl was activated by forming a pyridinium salt, which through a redox process with Cu^I^ produces pyridine, CuCl_2_ and a CF_3_SO_2_ radical, which easily underwent desulfonation to generate a CF_3_ radical. Finally, this radical was trapped by the alkenes to afford a new radical **3**, which after capture by CuCl_2_, gave a Cu^III^ species **7**. The final product **2** was obtained by reductive elimination and regeneration of the catalyst (Scheme 7). Additionally, the chlorotrifluoromethylation was suppressed by the radical scavenger TEMPO (2,2,6,6-tetramethylpiperidine 1-oxyl), which supports a radical mechanism.

**Scheme 8:**
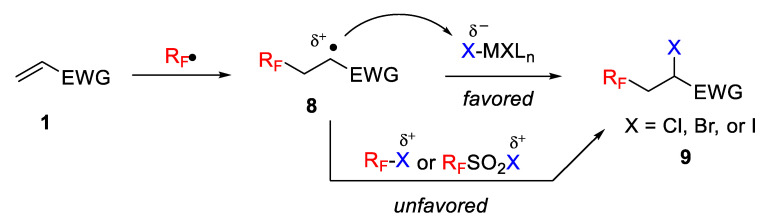
ATRA reaction of the fluoroalkyl radical with electron-deficient alkenes.

Atom transfer radical addition (ATRA) of R_F_X (X = I, Br) to electron-rich alkenes is a well-established methodology. However, until recently, the efficiency of this strategy had not been demonstrated for additions to electron-deficient alkenes due to the low conversion, selectivity, and yield obtained. The formation of undesired by-products and, particularly, the electrophilic nature of the radical intermediate formed during the process represent additional obstacles (Scheme 8). Thus, for example, due to their reducing ability, different Ru, Ir, and Cu species have not proved their efficiency in such reactions. In this context, Dolbier et al. recently studied photoinduced ATRA reactions of various fluoroalkyl-sulfonyl chlorides with electron-deficient alkenes to demonstrate the feasibility of this approach [36].

Previously, the same group had encountered that different fluorinated radicals such as R_f_ = CF_3_, CF_2_H, and CH_2_F, among others, could easily be generated from R_f_SO_2_Cl by photoredox catalysis under mild conditions as a way to obtain fluorinated 2-oxindoles by a tandem radical cyclization of *N*-arylacrylamides [37].

**Scheme 9:**
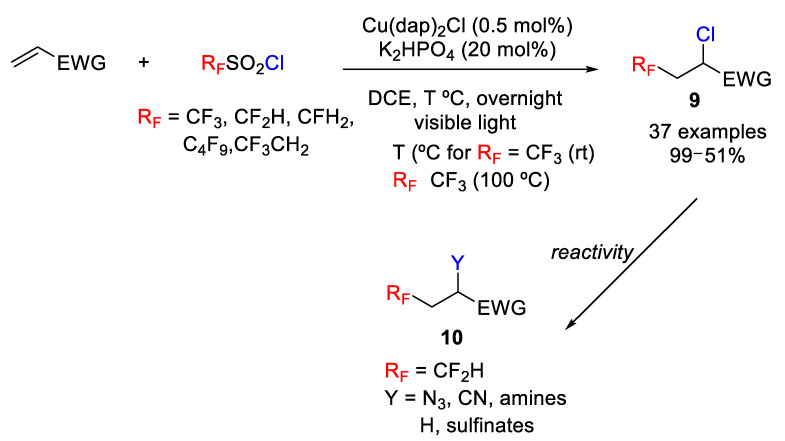
Reaction of R_f_SO_2_Cl with electron-deficient alkenes.

Dolbier first studied the reaction of CF_3_SO_2_Cl with electron-deficient alkenes using as a model system the α,β-unsaturated amide *N*-methyl-*N*-phenylacrylamide in the presence of different catalysts and reaction conditions. When Ru and Ir were used as catalysts either no reaction took place or low yields of products were obtained. The best results were achieved with Cu species, in particular, with the complex [Cu(dap)_2_]Cl [dap = 2,9-bis(4-methoxyphenyl)-1,10-phenanthroline] for which good chemical yields were obtained when the reaction was carried out at room temperature in either DCM or DCE as solvents. Finally, the addition of K_2_HPO_4_ (20 mol%) provided an almost quantitative yield of the target chlorotrifluoromethylation product **9**. Next, the scope of the process was studied with various electron-deficient alkenes such as α,β-unsaturated amides, esters, nitriles, and ketones under visible light using 0.5 mol% of [Cu(dap)_2_]Cl. Moderate to excellent yields were obtained in all cases studied (Scheme 9). In addition, CF_3_Cl was, in general, detected as by-product, attributed to a competitive abstraction of the chlorine atom from CF_3_SO_2_Cl or Cu(II) species by the intermediate trifluoromethyl radical.

The scope was extended to other previously obtained fluoroalkylsulfonyl chlorides (R_f_ ≠ CF_3_) with excellent results (Scheme 9). These findings are particularly interesting results due to the biologically importance of the CF_2_H group. In these cases, however, higher temperatures (100 °C) were required. Finally, the authors also explored synthetic applications of the target molecules. Thus, transformations such as the reduction, elimination, and substitution of the chlorine atom of the C-Cl bond by N_3_, CN, amine, and sulfinate were studied with high yields (Scheme 9).

**Scheme 10:**
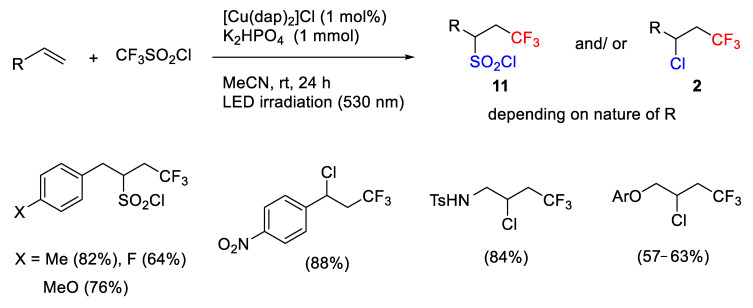
Cu-catalyzed trifluoromethylchlorosulfonylation vs trifluoromethylchlorination of alkenes.

Following this strategy, Reiser et al. also reported in 2015 an interesting study about the unprecedented trifluoromethylchlorosulfonylation of unactivated alkenes using the copper phenanthroline photoredox catalyst [Cu(dap)_2_]Cl under LED irradiation [38]. The authors provided evidence for an inner-sphere mechanism in contrast to other ruthenium, iridium and eosin Y catalyzed processes that give rise exclusively to trifluoromethylchlorination using the same alkenes. In the initial attempts, they observed, unexpectedly, that the green light irradiation (LED) of alkenes (using allylbenzene as a model system) with CF_3_SO_2_Cl in the presence of [Cu(dap)_2_]Cl and acetonitrile as solvent resulted in an ATRA process without SO_2_ extrusion providing the trifluoromethylchlorosulfonylation adduct **11** in moderate yield and only traces of the expected trifluoromethylchlorination product. Testing different reaction conditions such as catalysts, temperatures, and solvents showed that the presence of bases significantly improved the yield of **11**. Thus, the optimized conditions are the use of 1 mol% of [Cu(dap)_2_]Cl as catalyst, MeCN as solvent, addition of K_2_HPO_4_ (1 mmol), and LED irradiation (530 nm) (Scheme 10). Next, the scope of the process was examined, and the authors observed that allylbenzene with moderate donor or acceptor substituents on the aromatic ring give rise to excellent regioselectivity and good yields. Internal alkenes, however, provided mixtures of regio- and diastereoisomers. On the contrary, styrenes and substrates bearing donor atom in close proximity to the alkene functionality afforded the trifluoromethylchlorination product **2** (Scheme 10).

**Scheme 11:**
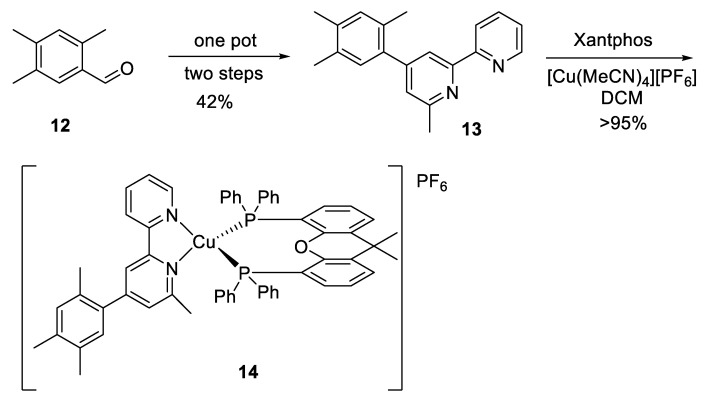
Synthesis of ligand **13** and complex **14**.

Despite the interest in the development of Cu-based photoredox catalysts, only a few Cu catalysts have been used in this kind of processes, with most of them containing one or two phenanthroline ligands. In this context, Xu and coworkers designed new copper complexes based on 4,6-disubstituted 2,2′-bipyridine ligands. In particular, the complex with Xantphos, [Cu^I^ L_n_ (Xantphos)][PF_6_], turned out to be an efficient catalyst for the chlorotrifluoromethylation of terminal alkenes, especially styrenes, which had not been suitable in this processes [39]. After testing several alternatives, it was concluded that complex **15** was the most efficient for this study. The synthesis of ligand **13** and complex **14** was prepared in three steps starting from 2,4,5-trimethylbenzaldehyde (**12**) by mixing ligand **13** with [Cu (MeCN)_4_][PF_6_] and Xantphos in DCM as solvent (Scheme 11).

**Scheme 12:**
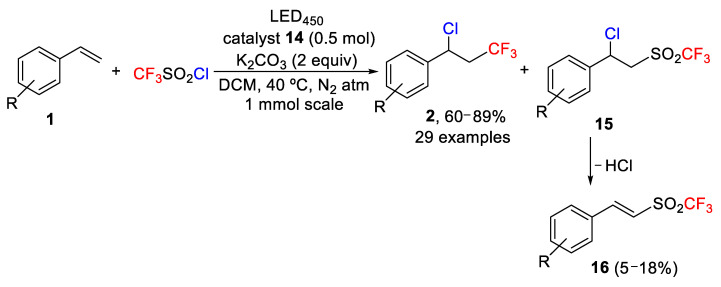
Chlorotrifluoromethylation with styrenes.

The efficiency of complex **14** as photoredox catalyst was tested in the chlorotrifluoromethylation of styrenes and CF_3_SO_2_Cl as trifluoromethyl source. The result of this study was the formation of two main products, the expected chlorotrifluoromethylation derivatives **2** and another **15** resulting from the chlorotrifluorosulfonation of the starting alkenes **16** (Scheme 12).

The process was very efficient with excellent conversion (100%) when the reaction was carried out at 40 °C using 1.2 equiv of CF_3_SO_2_Cl, 2 equiv of K_2_CO_3_ as a base, 0.5 mol% of catalyst in DCM and LED450 illumination. Under these conditions, a ratio of 15:1 was obtained in favor of chlorotrifluoromethylation product **2**. Other Ru, Ir, Cu, and organic photocatalysts provided less efficient results. Interestingly, when the LED source was replaced by sunlight, a similar conversion and selectivity were obtained. During this study, the authors found that chlorotrifluorosulfonation product **15** was sensitive to water and eliminates HCl during the isolation to yield compound **16**. The optimized conditions were successfully applied to a large number of substituted styrenes. Other aromatic groups such as naphthyl, pyridyl, and substrates derived from estrone, and α-tocopherol worked also well, proving the feasibility and tolerance of the method. The authors also studied the limitations of the process and found that internal alkenes were, in general, unreactive. In other cases, e.g., 4-vinylpyridine and 4-methoxy-1-vinylbenzene, polymerization was observed.

A mechanism was proposed based on previous studies with other terminal alkenes [17]. Thus, the photoexcited state of the Cu* catalyst undergoes oxidative quenching by CF_3_SO_2_Cl to give the CF_3_SO_2_ radical, which is trapped by the olefin, extruding SO_2_ to give a new CF_3_ radical, which adds to the olefin. The addition product is an alkyl radical able to take different reaction pathways [26].

**Scheme 13:**
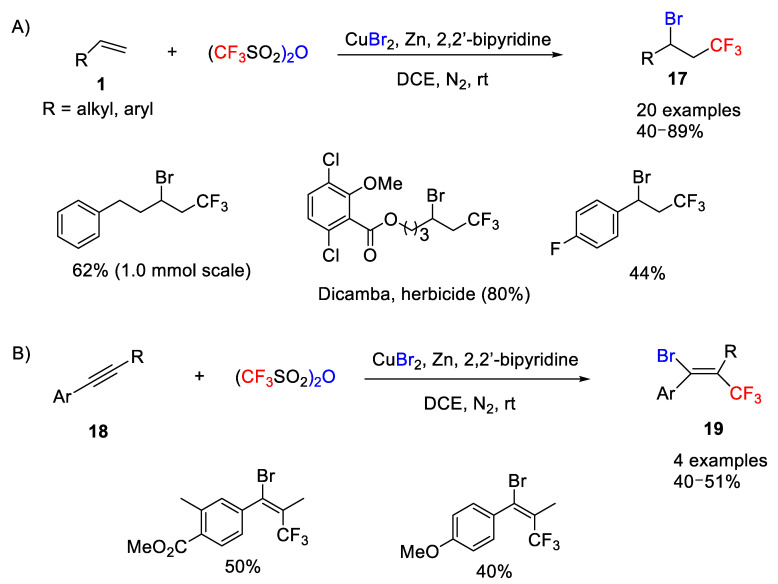
Cu/Zn promoted bromotrifluoromethylation of alkenes (**A**) and alkynes (**B**).

Very recently, Qing and co-workers reported the first copper/zinc-copromoted chloro- and bromo-trifluoromethylation of alkenes and alkynes using inexpensive and simple materials such as trifluoromethanesulfonic anhydride (CF_3_SO_2_)_2_O (Tf_2_O) [29,40] as trifluoromethylating reagent [41]. Thus, the reaction of alkenes and alkynes with Tf_2_O in the presence of copper salts such as CuX_2_ (X = Br, Cl), Zn powder and 2,2′-bipyridine provided the corresponding bromo- or chlorotrifluoromethylated products in good yields. The authors also showed the dual role that copper(II) salt plays in the process as catalyst and halogen source, whereas the 2,2′-bipyridine acts both as activating reagent of Tf_2_O and the ligand to coordinate to CuX_2_. With the optimized reaction conditions in hand, the scope of the copper/zinc copromoted process was studied. A variety of unactivated alkenes with a range of functional groups and styrenes with different substituents in the benzene ring were studied first, and the corresponding bromotrifluoromethylated derivatives **17** were obtained in good to excellent yields including biologically relevant compounds such as dicamba herbicide and estrone (Scheme 13A). Internal alkenes, however, turned out to be unsuitable substrates in this process. Moreover, this protocol is also suitable for terminal and internal alkynes **18**, which were converted into the substituted bromotrifluoromethylated alkenes **19** with good regio- and stereoselectivity and moderate yields (Scheme 13B).

**Scheme 14:**
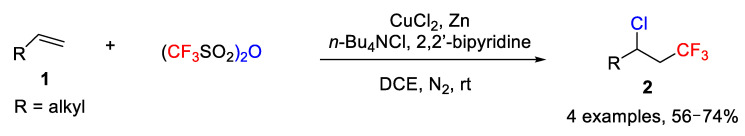
Cu/Zn promoted chlorotrifluoromethylation of alkenes with Tf_2_O.

This Cu/Zn-process was successfully extended to chlorotrifluoromethylation reactions by simply replacing the CuBr_2_ salt with CuCl_2_ using similar conditions as before. In this case, however, addition of *n*-Bu_4_NCl was necessary (Scheme 14). Unfortunately, this reaction could not be extended to fluoro- and iodotrifluoromethylation reactions.

**Scheme 15:**
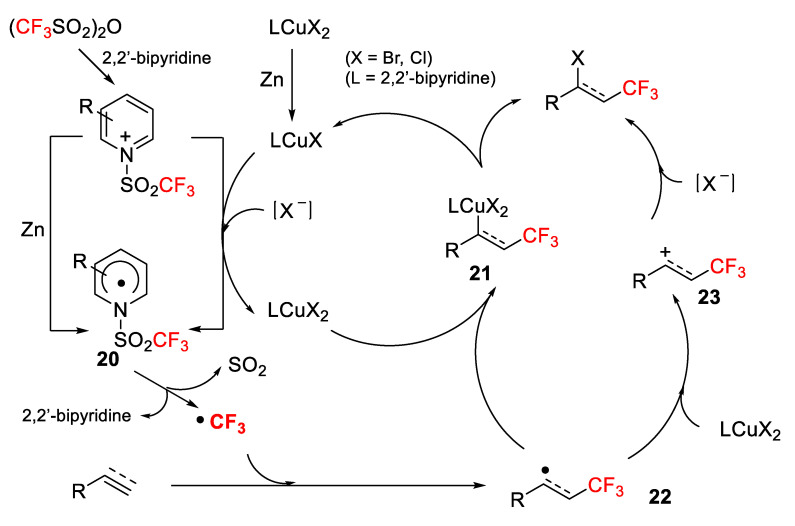
Proposed reaction mechanism.

The above results can be explained by the mechanism outlined in Scheme 15. First, the reaction of Tf_2_O and 2,2′-bipyridine (L) provides the *N*-triflylpyridinium salt, which after SET reduction with Zn powder affords a radical species **20** that collapses to liberate SO_2_, 2,2′-bipyridine and a CF_3_ radical. At the same time, LCuX_2_ (X= Cl, Br) is reduced by Zn powder to deliver LCuX, which acting as a reductant converts the *N*-triflylpyridinium salt into the radical species **20**. The addition of the CF_3_ radical to the alkene/alkyne gives a new β-trifluoromethyl radical **22**, which can progress through two possible pathways to the desired bromo/chloro final molecules (Scheme 15).

**Scheme 16:**
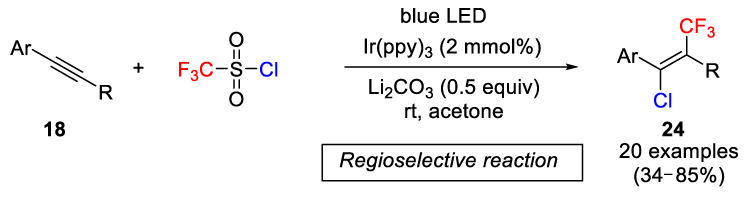
Chlorotrifluoromethylation of alkynes.

Following similar strategies but, in this case, carrying out the reaction under visible light catalyzed process, Han and coworkers described in 2017 a one-step regio- and stereoselective photoredox protocol for synthesizing tetrasubstituted alkenes by chlorotrifluoromethylation of internal arylalkynes **18** using CF_3_SO_2_Cl as both CF_3_ and chlorine source (Scheme 16) [42]. Tri- and, particularly, tetrasubstituted alkenes containing a CF_3_ group are described as useful intermediates in the synthesis of pharmaceutical and agrochemical agents [43]. After screening different ruthenium and iridium photocatalysts, bases and solvents, the optimal conditions were obtained with Li_2_CO_3_ as base, acetone as solvent and *fac*-Ir(ppy)_3_ (see, Figure 2) as photocatalyst. The alkyne scope of the process was studied under the optimized conditions, and it was found that the process takes place, in general, with moderate to good yields. A variety of functional groups and substituents in the arene ring worked efficiently. Thus, for example, alkynes containing acetyl, *p*-tosyl and Boc-protected amines in the arene ring, and 4-acetaminophenylalkynes containing various R groups at the nonaryl-substituted position provided moderate to good yields. The authors also highlighted that the reaction failed in the absence of visible light irradiation.

**Scheme 17:**
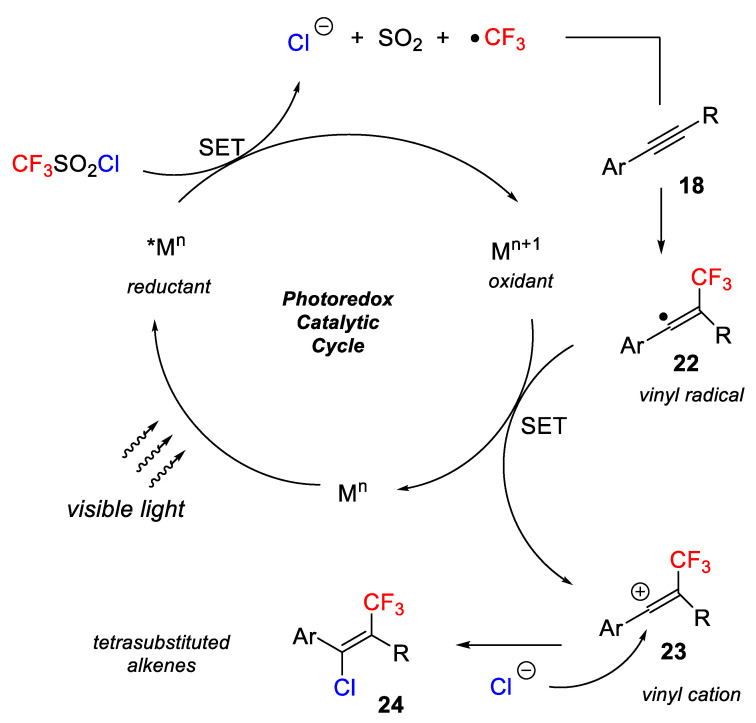
Proposed reaction mechanism for chlorotrifluoromethylation of internal aryl alkynes.

The above results were explained by the following mechanism: the excited state of the photoredox catalyst (*M^n^), generated by visible light, reacts CF_3_SO_2_Cl to provide a CF_3_ radical, SO_2_ and a chloride anion. The addition of the CF_3_ radical to the alkynes takes place regioselectively to produce an aryl ring stabilized the vinyl radical **22**. Next, the vinyl radical **22** is oxidized to generate a new vinyl cation **23** and the regeneration of the photocatalyst. The last step is addition of chloride to the vinyl cation **23**, finally yielding the target olefin **24**. The authors explained that due to electrostatic repulsion, the addition of the chloride takes place preferentially anti to the CF_3_ group (Scheme 17).

**Scheme 18:**
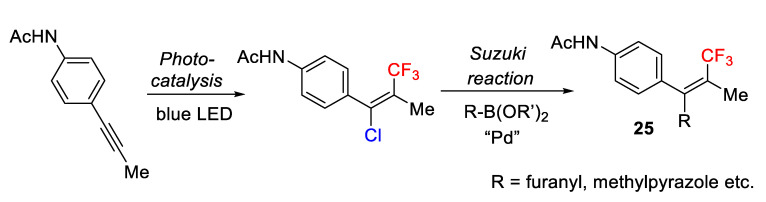
Pd-catalyzed Suzuki reaction.

The utility of this protocol for preparing more complex molecules was also studied in Suzuki coupling reactions with boronic acids. Thus, the chloro-atom was substituted, for example, by pyrazole, furan, and benzothiophene groups providing other interesting complex tetrasubstituted alkenes **25** (Scheme 18).

Notably, an interesting contribution concerning the chlorodifluromethylation and chlorocarbomethoxydifluoromethylation reaction of unactivated alkenes under metal-free conditions was also developed in 2015 by Dolbier et al. [44]. The incorporation of a CF_2_H, as well as a CF_2_CO_2_R group, is considered of particular interest in the pharmaceutical and agrochemical industry [45,46].

**Scheme 19:**
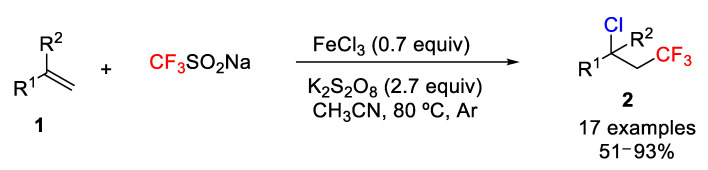
Iron-mediated chlorotrifluoromethylation of alkenes with Langlois reagent.

A different and practical strategy for the iron-mediated chlorotrifluoromethylation of styrenes and aliphatic alkenes was presented by the Qing group in 2016 [47]. The process uses inexpensive and solid materials such as ferric chloride (FeCl_3_) as the Cl source and the Langlois reagent [48] (sodium triflinate, CF_3_SO_2_Na) as the CF_3_ source. After screening different metal salts, solvents, and temperatures, the authors found that FeCl_3_ and MeCN as salt and solvent, respectively, were the best option. Other salts (Cu^+1^and Cu^+2^) and solvents (DMF, DMSO, etc.) were less efficient (Scheme 19). The scope of the iron-mediated chlorotrifluoromethylation of alkenes showed that styrenes bearing electron-donating and electron-withdrawing groups reacted smoothly as well as unactivated aliphatic alkenes to give the target molecules **2** in good to excellent yields. This protocol was also efficient for biologically active compounds such as vinyl-*N*-benzoyl-*L*-tyrosine, showing that this method could be suitable for the “late-stage chlorotrifluoromethylation” of natural products and drugs.

**Scheme 20:**
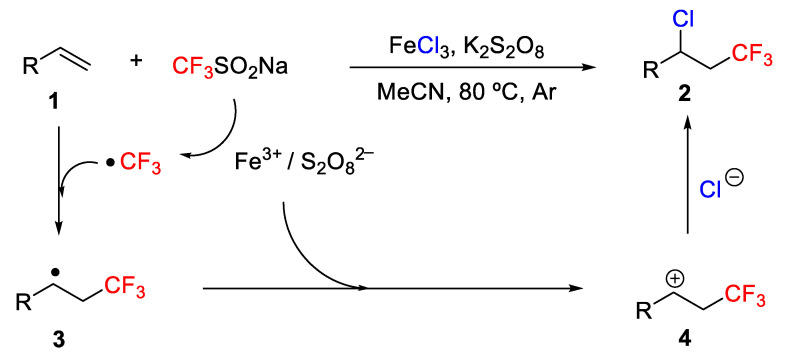
Proposed mechanism.

The mechanism of the process was studied. Thus, a trifluoromethylradical is generated from Langlois reagent through a SET process under the treatment of FeCl_3_ and K_2_S_2_O_8_, which then reacts with the alkene to give the radical intermediate **3**. Finally, the radical intermediate **3** was oxidized by FeCl_3_ and K_2_S_2_O_8_ to generate a cation **4**, which undergoes a nucleophilic attack by the chloride anion to provide the final addition product (Scheme 20). In the presence of TEMPO, the reaction was inhibited, suggesting a radical/cationic process.

**Scheme 21:**
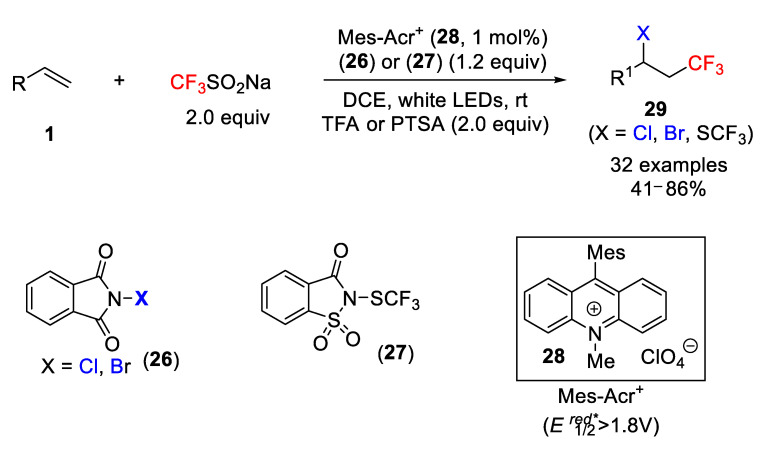
Halo- and SCF_3_-trifluoromethylation of unactivated alkenes.

The easy-handling Langlois reagent has also been used successfully in photoredox processes. Thus, quite recently, Feng Liu and coworkers reported a mild, one-pot, and transition-metal-free photoredox halo- and trifluoromethylthio-trifluoromethylation of simple alkenes with sodium triflinate [49]. In this process, *N*-chloro- and bromophthalimide (**26**) and *N*-trifluoromethylthiosaccharin (**27**) were used as halogen and SCF_3_ sources, respectively. An organic photoredox catalyst, 9-mesityl-10-methyl acridinium (Mes-Acr^+^) perchlorate (**28**), with an excited-state reduction potential of 1.8 V, was used under visible-light irradiation (white LED). The authors found that the optimized conditions are the use of 1,2-dichloroethane (DCE) as a solvent, TFA (trifluoroacetic acid) as acid additive, Langlois reagent as CF_3_ source, *N*-chloro- (NCP) and *N*-bromoimides (NBP) as halo source, and Mes-Acr^+^ as photocatalyst. It was also found that irradiation with blue LED gave satisfactory results. The scope of the process was studied, and it became apparent that many functional groups including amides, ketones, imides, esters, etc., were tolerated and smoothly converted into the desired final products **29** with high yields. The case of 2,2-diallylmalonate for which a radical cascade took place is particularly interesting. Simple control experiments with TEMPO supported the presence of radicals in the process. This protocol was extended to the trifluoromethylthiotrifluoromethylation of simple alkenes using *N*-trifluoromethylthiosaccharin **27** as the SCF_3_ transfer reagent with similar results. In this case, however, *p*-toluensulfonic acid (PTSA) was the best acid additive (Scheme 21).

**Scheme 22:**
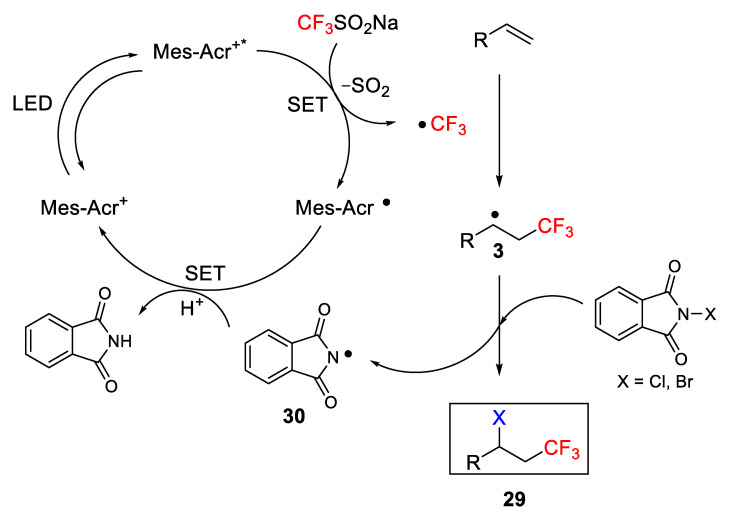
Proposed mechanism for the halotrifluoromethyation of alkenes.

A plausible mechanism was proposed to rationalize the formation of the desired product. According to the authors, the process was initiated by a single electron transfer of the Langlois reagent by the photoexcited Mes-Acr^+^* generating a CF_3_ radical and an acridine radical. The subsequent addition of the CF_3_ radical to the olefin followed by halogenation with NXS (X = Cl, Br) lead to the final product **29** (Scheme 22).

**Scheme 23:**
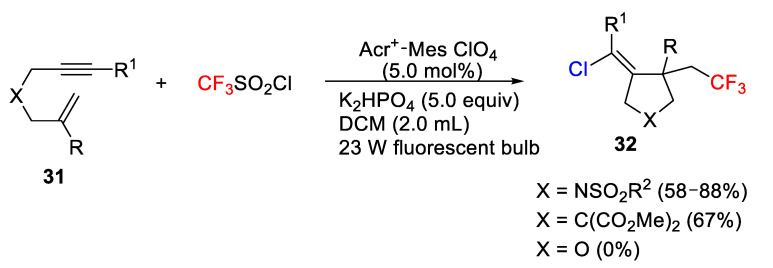
Chlorotrifluoromethylative and chlorotrichloromethylative cyclization of enynes.

Following a similar strategy but using enynes instead of simple olefins, a cascade cyclization of enynes was recently reported by Hou, Chen, and Zhu’s group. Thus, a new visible-light-mediated photocatalytic chlorotrifluoromethylation and chlorotrichloromethylation of 1,6-enynes **31** in the presence of CX_3_SO_2_Cl (X= F, Cl) is described [50,51]. The process proceeds through an initial radical addition, followed by a cyclization/chlorination sequence. The use of terminal alkene-derived 1,6-enynes provided an array of carbo- and heterocycle derivatives **32** in a regio- and stereoselective manner (Scheme 23). In this process, both Mes-Acr^+^ ClO_4_^−^ and Ir(dtbbpy)(ppy)_2_PF_6_ were effective catalysts showing that the transition metal was not essential. Although different Ir and Ru complexes were tested, the best result was obtained when the organic dye Acr^+^-Mes ClO_4_ was used as catalyst and DCM as solvent under irradiation with a 23 W fluorescent bulb. Although the process worked quite well for nitrogen derivatives (X = NSO_2_R^2^) and all-carbon cyclic compounds, the method failed for oxygen derivatives (X = O).

**Scheme 24:**
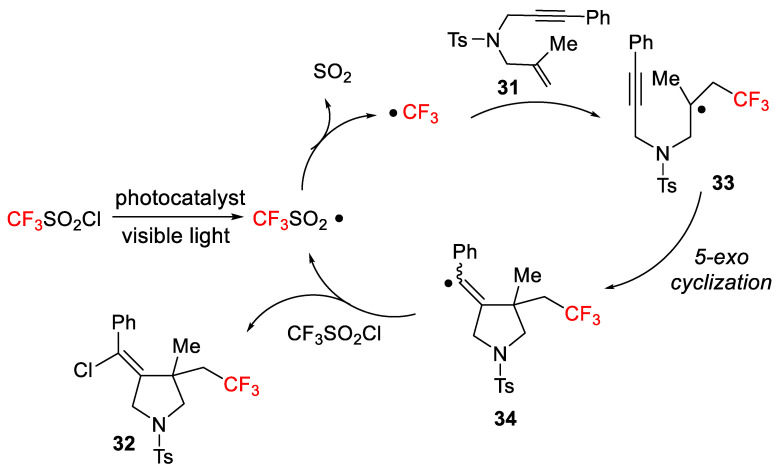
Mechanism for the chlorotrifluoromethylation of enynes.

The mechanism of the process for the chlorotrifluoromethylation is based on previous reports and it is outlined in Scheme 24. Once the CF_3_ radical is generated, its addition to the enyne double bond provides a tertiary carbon radical intermediate **33**. The 5-*exo* radical cyclization results in a new vinyl radical **34** formation, which finally reacts with CF_3_SO_2_Cl affording through a chlorine atom transfer reaction the final *Z*-products **32** and regeneration of the sulfonyl radical. Interestingly, the 6-*endo* radical cyclization was not observed.

**Scheme 25:**
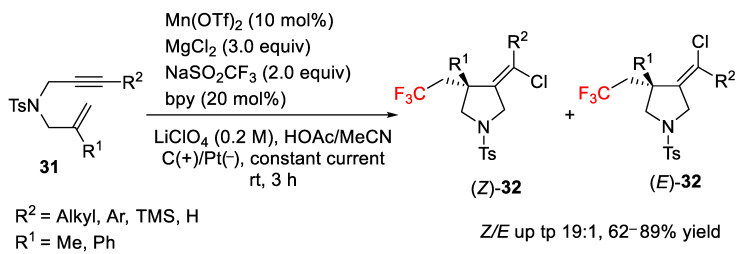
Electrocatalytic synthesis of pyrrolidine derivatives.

Previously, Song Lin et al. [52] had reported a convenient procedure for the preparation of chlorotrifluoromethylated pyrrolidine derivatives, which are interesting molecules in medicinal chemistry research, by electrocatalytic radical ene-yne cyclization. The process was carried out using anodically coupled electrolysis and the incorporation of a chelating ligand such as 2,2′-bipyridine (bpy) provided the final products **32** with high regio- and stereochemical control in favor of the (*Z*)-isomer. The Langlois reagent (CF_3_SO_2_Na) and MgCl_2_ were used as CF_3_ radical and Cl^−^ sources via electrochemical oxidation as well as different manganese salts as catalysts. After checking different reaction conditions, it was found that the use of Mn(OTf)_2_ as a catalyst, a mixture of HOAc/MeCN as solvent, MgCl_2_ and CF_3_SO_2_Na salts as CF_3_ radical and Cl^−^ sources, and the application of a constant current of 10 mA were the most efficient conditions in terms of chemical yield (up to 89%) and selectivity (*Z/E* > 19:1). Interestingly, the addition of bpy as ligand increased the yield and selectivity notably. When the reaction was carried out in absence of the Mn catalyst, no product was obtained indicating the crucial role of the catalyst in the process (Scheme 25). Concerning the scope, aryl, alkyl, silyl, and terminal alkynes were also compatible with the described electrocatalytic conditions.

**Scheme 26:**
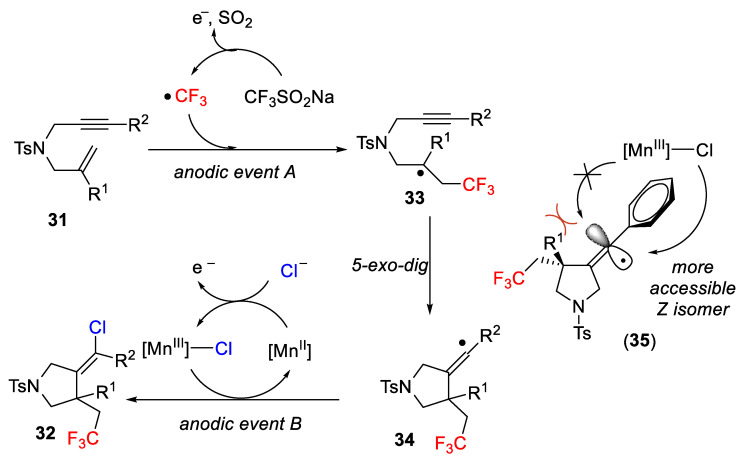
Proposed mechanism and stereochemistry.

The proposed mechanism of the process is outlined in Scheme 26. The starting 1,6-substrates undergo a cascade reaction, which is a selective trifluoromethylation, radical cyclization (*5-exo-dig cyclization*) and final chlorination to yield the target pyrrolidines **32**. The mechanism entails the initial simultaneous anodic oxidation of the Langlois reagent (formation of the electrophilic radicals CF_3_, anodic event A) and [Mn^III^]-Cl (anodic event B, formed by mixing the Mn catalyst and MgCl_2_). The final *Z/E*-stereochemistry is sterically controlled by the conjugation between the aryl π-orbital and the single occupied molecular orbital in intermediate (**35**). This orbital is shielded by the substituents at the quaternary carbon α, favoring the approach on the less congested side to the final *Z*-isomer.

**Scheme 27:**
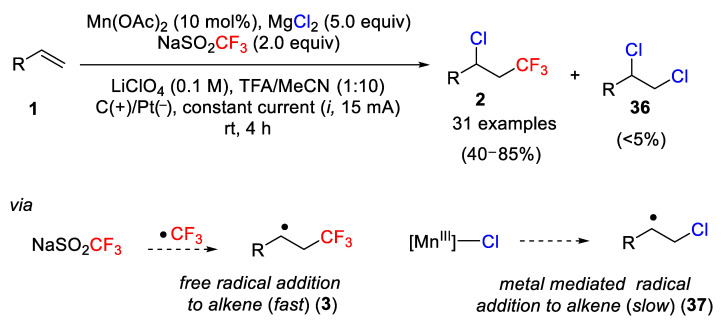
Heterodifunctionalization of alkenes by anodic coupled electrolysis.

Using a similar strategy, the same group had simultaneously developed a new protocol based on the anodically coupled electrolysis for the regio- and chemoselective chlorotrifluoromethylation of simple alkenes [53]. The optimized reaction conditions were studied, and it was found that the Langlois reagent and MgCl_2_ were the starting materials of choice to carry out the process successfully. The most favorable reaction conditions are outlined in Scheme 27. Thus, Mn(AcO)_2_ + MgCl_2_ generate the latent radical chlorination agent [Mn^III^]-Cl, trifluoroacetic acid (TFA) as the sacrificial oxidant and proton source, LiClO_4_ as the electrolyte in MeCN, and a constant current of 15 mA were the most efficient conditions found.

The scope of the process was also investigated, and it was found that cyclic and aliphatic alkenes and a variety of styrene derivatives with oxidative labile functional groups were compatible with those conditions. However, electron-rich styrenes were not effective in the process. Furthermore, small amounts of the corresponding dichloride derivatives as undesired compounds were also detected (Scheme 27). This methodology was also applied to the difunctionalization of alkynes and the bromotrifluoromethylation of alkenes using, instead of MgCl_2_, potassium bromide (KBr) as the bromine source (Scheme 27). The mechanism of the process is similar to that described by the same group (see Scheme 26), but using simple alkenes instead of 1,6-enynes as starting materials. Radical intermediates such as **3** (free radical addition to the alkene, fast) and **37** (metal-mediated radical addition to the alkene, slow) explained the obtained results (Scheme 27).

**Scheme 28:**
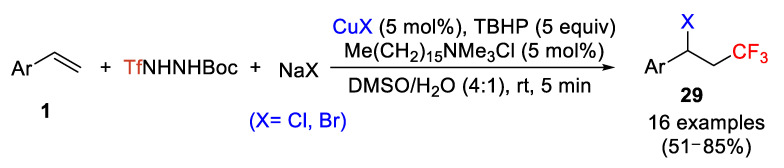
Chlorotrifluoromethylation of arylalkenes.

In the chlorotrifluoromethylation of alkenes, several reagents (in particular, CF_3_SO_2_Cl and others) have been reported for that purpose. However, most of them suffer some problems in terms to find mild reaction conditions. An unprecedented and relatively new strategy makes use of the solid and bench-stable trifluoromethanesulfonyl hydrazides as trifluoromethylating reagent for the vicinal difunctionalization of terminal alkenes [54]. Thus, Tian et al. reported in 2016 that TfNHNHBoc, prepared by reacting triflic anhydride with monosubstituted hydrazines, and NaCl are efficient substrates for the vicinal chlorotrifluoromethylation of styrene derivatives in the presence of CuCl as redox catalyst. The use of Me(CH_2_)_15_NMe_3_Cl as a phase transfer catalyst, DMSO/water (4:1) as solvent at room temperature, and TBHP (*tert*-butyl hydroperoxide) as oxidant provided the most effective conditions. Excellent regioselectivity and good chemical yield were, in general, obtained. This process was extended to the bromotrifluoromethylation of arylalkenes by simple substitution of NaCl and CuCl by NaBr and CuBr increasing the scope of the process (Scheme 28). On the other hand, the process failed in the iodotrifluoromethylation of arylalkenes.

**Scheme 29:**
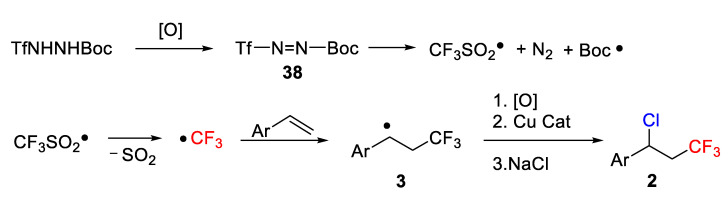
Proposed mechanism for the chlorotrifluoromethylation reaction with TfNHNHBoc.

A plausible mechanism of the process is depicted in Scheme 29. Thus, oxidation of TfNHNHBoc leads to the formation of a diazene **38**, which decomposes into a CF_3_ radical with extrusion of SO_2_. Regioselective addition to the alkene followed by copper-catalyzed oxidation, formation of a carbocation and reaction with Cl^−^ gave the final product **2**.

**Scheme 30:**
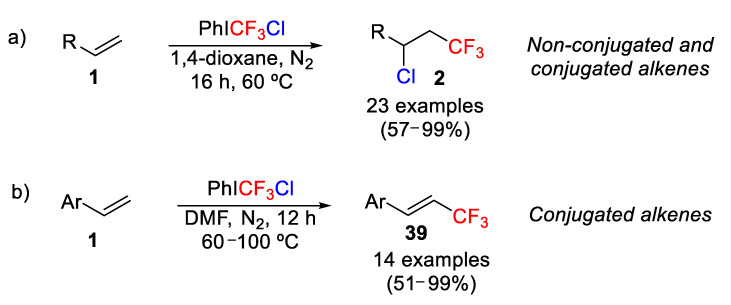
Uncatalyzed halo- and trifluoromethylation of non-conjugated alkenes (**a**) and conjugated alkenes (**b**).

In 2019, Wang and coworkers reported the use of the CF_3_-containing λ^3^-iodane, PhICF_3_Cl [55], as a bifunctional reagent for the uncatalyzed chlorotrifluoromethylation of non-conjugated and conjugated alkenes [56]. This process was also successfully extended to biologically important molecules such as ibuprofen, indomethacin, estrone, and oxaprozin derivatives among others. The best reaction conditions are the use of 1,4-dioxane as solvent, nitrogen atmosphere, and temperatures of 60 °C (Scheme 30a). Moreover, the nature of the final product is highly dependent on the reaction conditions. Thus, when the reaction was carried out at higher temperatures (60–100 °C) and the solvent was DMF, the obtained products were identified as vinyl derivatives, being the result of the elimination of HCl of the initially formed chlorotrifluoromethylated product. This “side” reaction was particularly effective in the case of the styrene derivatives (Scheme 30b).

**Scheme 31:**
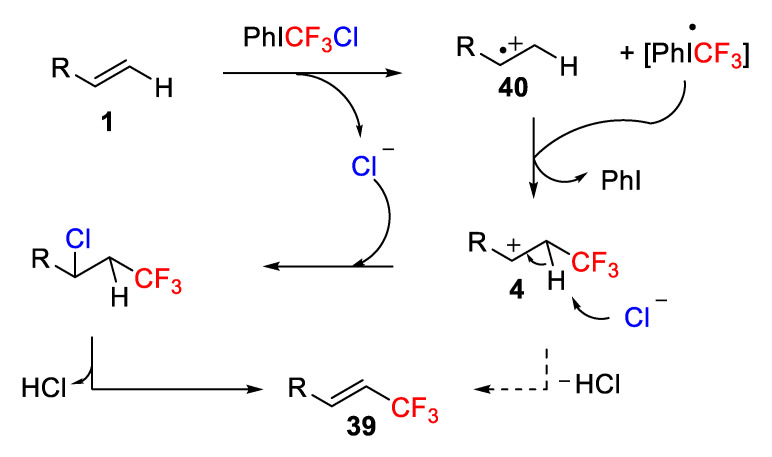
Radical cation pathway.

A plausible mechanism was proposed for this process. Thus, the initial reaction of the alkene with PhICF_3_Cl undergoes a SET process to generate a radical cation and a hypervalent iodine radical, followed by a coupling of the radical cation **40** with the generated CF_3_ radical affording a new carbocation **4** and releasing of PhI. Final nucleophilic attack on the chloride anion to the carbocation provided the target molecule **39** (Scheme 31). In this mechanism, an ionic pathway cannot be ruled out. Finally, vinyl C-H trifluoromethylation products were obtained by elimination of HCl in the case of conjugated alkenes.

**Scheme 32:**
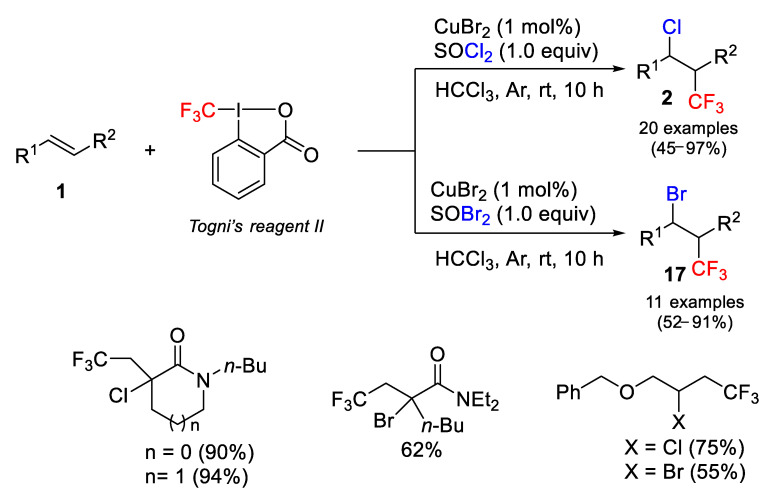
Copper-catalyzed intermolecular halotrifluoromethylation of alkenes.

Although the halotrifluoromethylation of alkenes has recently been studied in some detail, complex reaction conditions are required in most of the cases, including expensive photoredox catalysts (Ru, Ir and Cu complexes), high temperatures and the use of sophisticated bases. In this context, Jiang, Yang, and co-authors [57] described a general, gram scale, three-component and efficient method for the intermolecular chloro- and bromotrifluoromethylation of electron-deficient and unactivated alkenes using simple CuBr_2_ as catalyst and Togni reagent in combination of dihalo-sulfoxide (SOX_2_, X = Cl, Br) as the trifluoromethylation and halogen sources, respectively. The optimized and mild reaction conditions are the use of CHCl_3_ as solvent, room temperature, catalyst loading of 1 mol%, 1.5 equiv of Togni II reagent, and 1.0 equiv of SOX_2_. The scope of the process is wide providing good to excellent results with cyclic pyrrolidinon derivatives, linear α,β-unsaturated amides as well as simple aliphatic alkenes. Interestingly, Umemoto reagent and Togni reagent I were either inactive or much less efficient in the process. In the case of the bromoderivatives, PBr_3_ was used as bromine source in some cases instead of thionyl bromide (Scheme 32). Control experiments showed that both the copper catalyst and the halo-source were essential for the success of the process. When the reaction was carried out in the presence of TEMPO, no final product was obtained indicating the radical nature of the process.

**Scheme 33:**
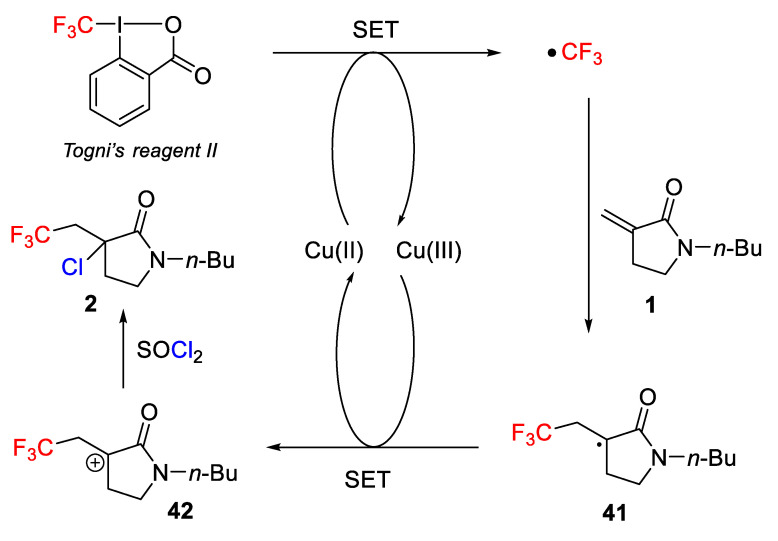
Proposed reaction mechanism.

Considering the obtained results, a simple mechanism was proposed (Scheme 33). Togni reagent is activated by the copper catalyst, generating a radical CF_3_ (SET process) and release of a Cu^+3^ species. The CF_3_ radical was then added to an electron-deficient alkene generating a new radical **41**, which underwent a second SET process, affording a carbocation **42** and regeneration of the active Cu^+2^ catalyst. Finally, the formed carbocation is attacked by Cl^−^ anion of the thionyl chloride.

**Scheme 34:**
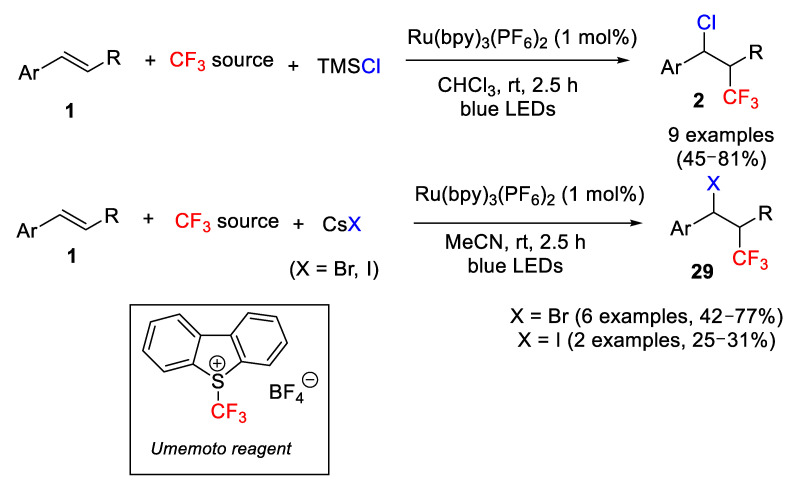
Photoredox-mediated chloro-, bromo-, and iodotrifluoromethylation of alkenes.

Although many reports have been published using different strategies and trifluoromethyl sources for the chlorotrifluoromethylation of olefins in the last decade, the same does not apply to the other halogens. In fact, very few protocols have been established for the general halotrifluoromethylation of alkenes, and a lot less in the case of styrene derivatives. In this sense, Masson et al. [58] described in 2015 a quite general protocol for the three-component halotrifluoromethylation of olefins in a photoredox process catalyzed by the Ru(bpy)_3_(PF_6_)_2_ complex as photosensitizer and Umemoto reagent as CF_3_ source. Concerning the nature of the halogen, the process can be applied to chloro-, bromo-, and iodo-derivatives, and the described method is particularly efficient in the case of styrene derivatives for which moderate to good yields were obtained. Moreover, short reaction times and mild reaction conditions were required. In the case of the chloro-derivatives, TMSCl was used as chlorine source and HCCl_3_ as solvent. These conditions failed for the other halogens for which a CsX (X = Br, I) salt as halogen source and MeCN as solvent were used (Scheme 34).

**Scheme 35:**
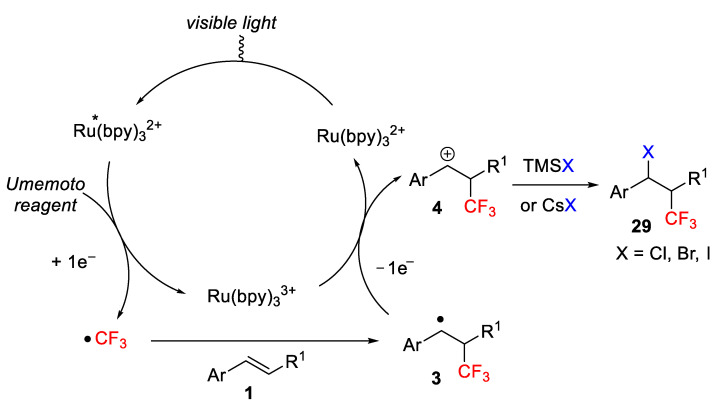
Proposed mechanism for the halotrifluoromethylation of alkenes.

A reasonable mechanism for this multi-component process is shown in Scheme 35. The process is initiated by the formation of a strong reductant species *Ru(bpy)_3_^2+^, obtained from the photoredox initiator after excitation with visible light. Then, a single electron transfer generates an electrophilic CF_3_ radical from Umemoto reagent, which next reacts with the olefin to give an α-aryl radical intermediate affording a new α-aryl carbocation. Final nucleophilic trapping of the carbocation by Cl^−^, Br^−^, or I^−^ leads to the trifluoromethylated product **29**.

**Scheme 36:**
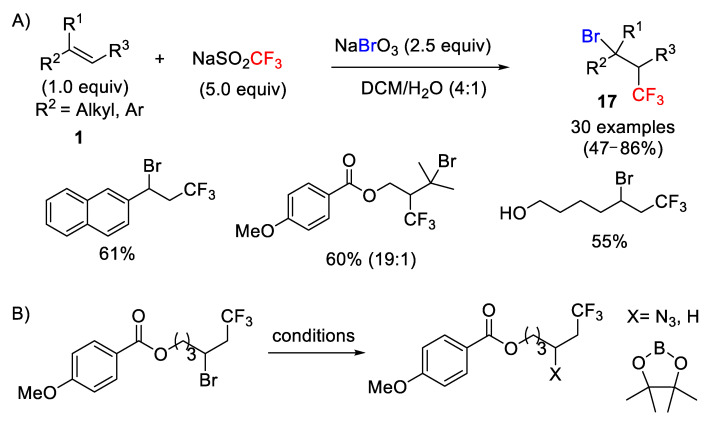
NaBrO_3_-promoted bromotrifluoromethylation of styrenes and unactivated alkenes (**A**); utility of the obtained product **17** (**B**).

In 2014, Liu and co-authors described an effective and general methodology for the iodotrifluoromethylation of alkenes and alkynes using the Langlois reagent and iodine pentoxide (I_2_O_5_) as CF_3_ and iodine sources [59]. More recently, the same group extended this methodology to the bromotrifluoromethylation of unactivated olefins and styrenes using an appropriate bromine source. After having screened several bromides, they found that sodium bromate (NaBrO_3_) along with the Langlois reagent were the most efficient reagents [60]. The metal-free process worked particularly well when a mixture of DCM/H_2_O (4:1) as solvent was applied. It is noteworthy that sodium bromate plays a dual role in the process acting both as a single-electron oxidant and a bromine source. Under these conditions, a wide range of unactivated alkenes with different functional groups, including nonterminal alkenes and styrene derivatives, were well tolerated and, in general, moderate to good yields were obtained (Scheme 36A). The authors also demonstrated that a large-scale (gram level) was also possible, thus increasing the potential of this methodology in the chemical industry as well as the utility of the obtained molecules in synthetic organic chemistry (Scheme 36B).

**Scheme 37:**
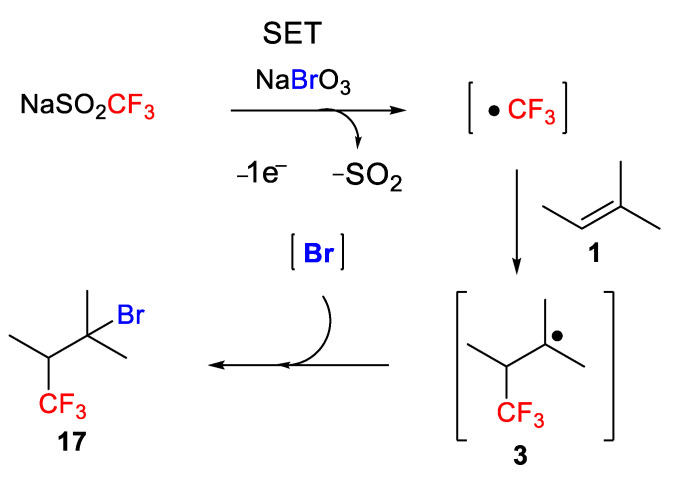
Suggested mechanism in the metal-free bromotrifluoromethylation of alkenes.

The formation of the target molecules was initiated by a single-electron oxidation of the Langlois reagent by NaBrO_3_ to generate a CF_3_ radical. Next, the CF_3_ radical adds to the double bond to afford a new radical intermediate **3**, which finally undergoes the bromination step to result in the target product **17** (Scheme 37). Different mechanistic studies involving a radical-clock experiment and electron-spin resonance (ESR) detection supported the radical nature of the process.

**Scheme 38:**
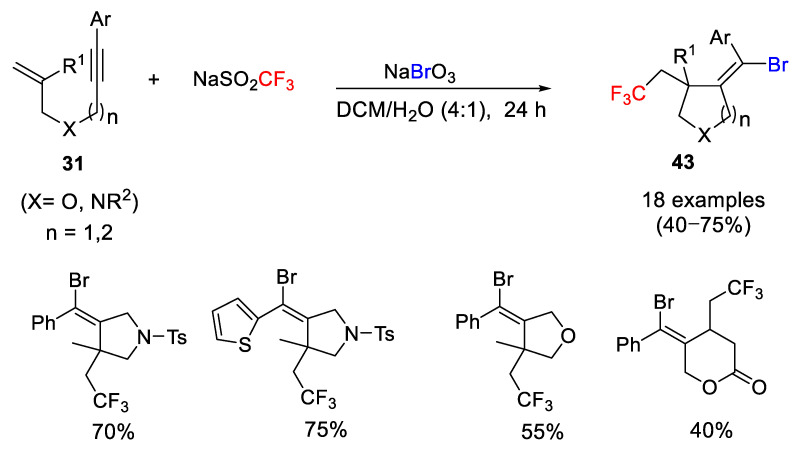
NaSO_2_CF_3_/ NaBrO_3_-promoted bromotrifluoromethylation of enynes.

In order to extend the utility of this methodology, the same group studied, one year later, the behavior of 1,6- and 1,7-enynes in this kind of processes. The result was, using the same reaction conditions (NaSO_2_CF_3_/ NaBrO_3_), the facile access to a wide range of 5- and 6-membered heterocycles bearing a CF_3_ group and Br atoms in their framework (Scheme 38). This free-radical cascade process took place under mild metal-free reaction conditions with good chemical yields and high chemoselectivity [61].

**Scheme 39:**
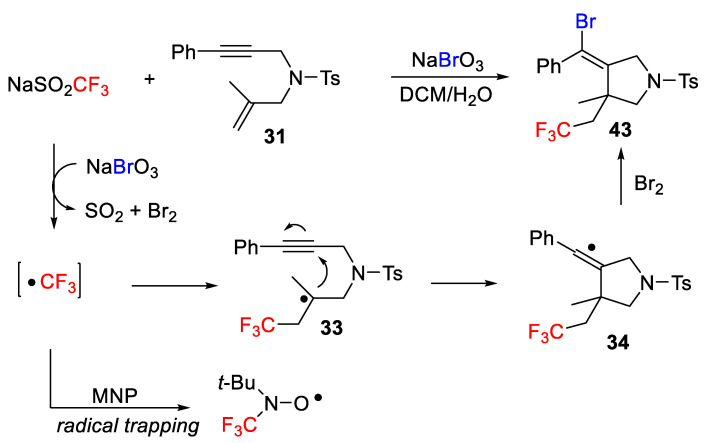
Proposed mechanism and radical trapping.

The mechanism of the process is quite similar to the one previously described for alkenes (see Scheme 37). Initially, a CF_3_ radical is generated by electron transfer from NaSO_2_CF_3_ to NaBrO_3_ followed by release of SO_2_ and Br_2_. Next, radical addition and cyclization afford a new vinyl radical. Final Br-atom abstraction completes the process (Scheme 39). Interestingly, the CF_3_ radical was trapped by 2-methyl-2-nitropropane (MNP), which demonstrated the radical nature of the process.

**Scheme 40:**
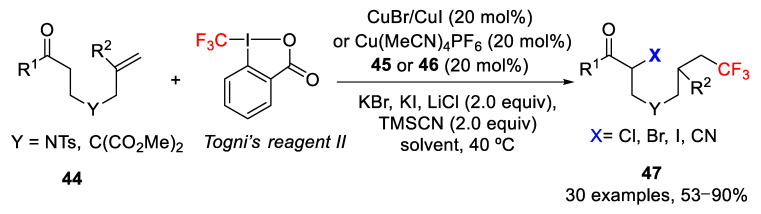
Remote halo- and cyanotrifluoromethylation of carbonyl compounds.

A completely different and novel strategy was reported by Tan, Liu, et al. for the radical tandem trifluoromethylation-initiated remote cross-coupling of a variety of carbonyl compounds containing an alkene moiety **44** with simple nucleophiles such as halogens (Cl, Br, I) and a cyano group, using Togni reagent II as the CF_3_ source (Scheme 40) [62]. The process provided the simultaneous formation of two new C-CF_3_ and C-X (X= Cl, Br, I, CN) bonds, in which the key step is an intramolecular 1,5-H radical shift. After checking different reaction conditions, the best results were obtained in the case of the remote bromo-trifluoromethylation with CuBr as catalyst, KBr as inorganic salt, 2,2′-bipyridyl (**45**) as ligand and EtOAc as a solvent. Similar reaction conditions were employed for chloro- and iodo-trifluoromethylation, but in this case using CuI as catalyst and inorganic salts such as LiCl or KI, respectively. In order to extend this methodology, the authors also studied the remote α-cyano-trifluoromethylation of carbonyl compounds containing an alkene in the ω-position. In this latter case, the optimal conditions are the use of the Cu(MeCN)_4_PF_6_ complex as catalyst, 1,10-phenanthroline (**46**) as ligand, trimethylsilyl cyanide (TMSCN) as cyano source and MeCN as solvent. The process worked well with a variety of aliphatic and aromatics alkenyl carbonyl derivatives, including ketones, esters, amides, etc. In all cases, moderate to good yields were obtained and the final products **47** were easily transformed into valuable CF_3_-containing aza-heterocycles.

**Scheme 41:**
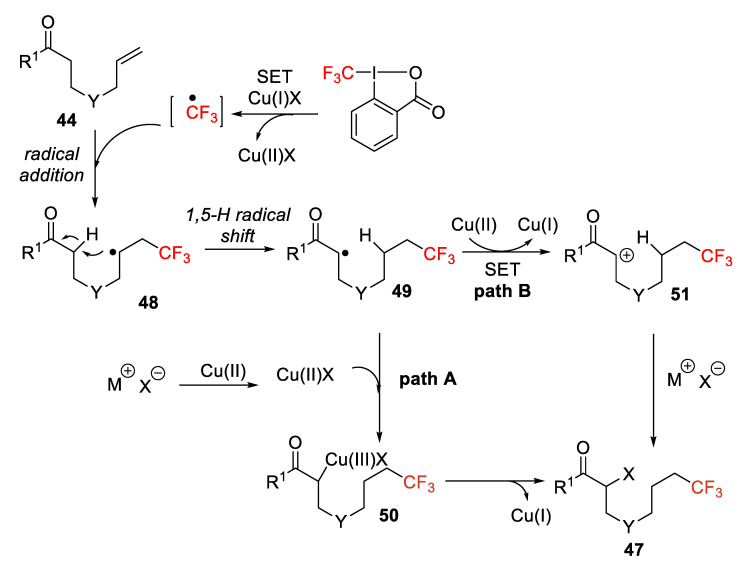
Proposed mechanism for the remote halo-and cyanotrifluoromethylation of alkenes.

After several control experiments including reactions with TEMPO or 2,6-di-*tert*-butyl-4-methylphenol (BHT) and with deuterated compounds, the authors concluded that the process proceeds through an intramolecular 1,5-H radical shift. Thus, the CF_3_ radical generated from the reaction of Togni reagent with Cu(I) attacks the alkene moiety providing intermediate **48**, followed by a 1,5-H radical shift obtaining radical **49**, from which a new Cu(III) species **50** originates. Finally, reductive elimination leads to the target product (path A). Another alternative is the formation of carbocation **51** by single-electron oxidation with Cu(II) species and nucleophilic addition of inorganic halogen salt or TMSCN (path B) (Scheme 41). This strategy provides a novel and elegant procedure for synthesizing trifluoromethyl α-halo and α-cyano-carbonyl derivatives of great importance in the pharmaceutical industry.

## 3. Iodotrifluoroalkylation

Among trifluoromethylation-involved difunctionalization of unsaturated compounds, the iodotrifluoromethylation of alkenes and alkynes, has attracted much attention in the past decade [63]. However, most all reported strategies for iodotrifluoromethylation of olefins and alkynes are based on using trifluoroiodomethane (CF_3_I) as the trifluoromethylation source, which is a gas and hampers the handling and operation. Consequently, operationally safer, and more sustainable iodotrifluoromethylation of alkenes and alkynes have emerged as alternative methods for the same purpose.

In this regard, different iodotrifluoromethylation methods have been developed including visible-light-induced approaches based on the use of transition-metal complexes as photosensitizers [64]. Most of these approaches are based on the use of iridium and ruthenium photocatalysts as they have efficiently catalyzed the formation of carbon-fluoroalkyl bonds under visible light irradiation [65]. Photoredox catalysts based on ruthenium or iridium metal complexes from polybipyridyl ligands absorb blue light, and high yields were generally achieved with high chemoselectivities under mild reaction conditions.

**Scheme 42:**
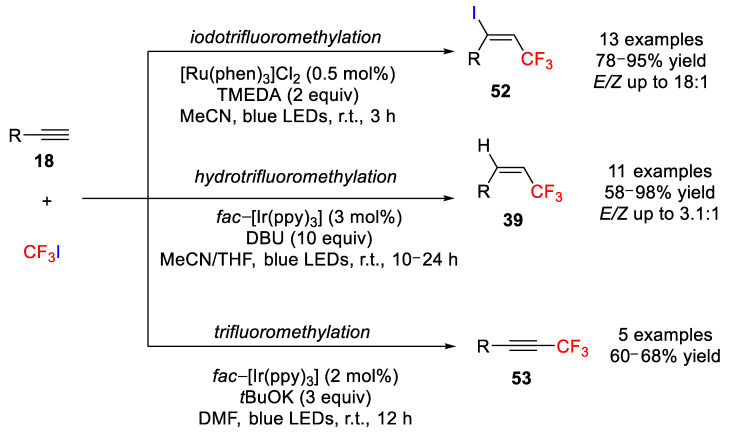
Controlled trifuoromethylation reactions with CH_3_I under blue-light irradiation with iridium and ruthenium photocatalysts.

Using this approach, in 2014, Cho and coworkers developed a controlled methodology based on the use of iridium and ruthenium photocatalysts for the iodotrifluoromethylation and hydrotrifluoromethylation of alkynes **18** (Scheme 42) [66]. The authors initially examined the iodotrifluoromethylation of terminal alkynes with CF_3_I and *N*,*N*,*N*’,*N*’-tetramethylethylenediamine (TMEDA) in MeCN under visible light irradiation (blue LEDs) in the presence of a wide range of iridium and ruthenium photocatalysts, including *fac*-[Ir(ppy)3], [Ir(ppy)_2_(dtb-bpy)]PF_6_, [Ru(bpy)_3_]Cl_2_, and [Ru(phen)_3_]Cl_2_. Despite all catalysts favoring the generation of the iodotrifluoromethylation product in high yields and high *E/Z* ratios (from 17:1 to 20:1), [Ru(phen)_3_]Cl_2_ was chosen as the optimal catalyst for evaluating the scope of iodotrifluoromethylation because of its low cost and simplicity of reaction. In this study, the iodotrifluoromethylation of a variety of aromatic and aliphatic alkynes bearing several functional groups (13 examples) was accomplished with excellent *E/Z* stereoselectivities.

**Scheme 43:**
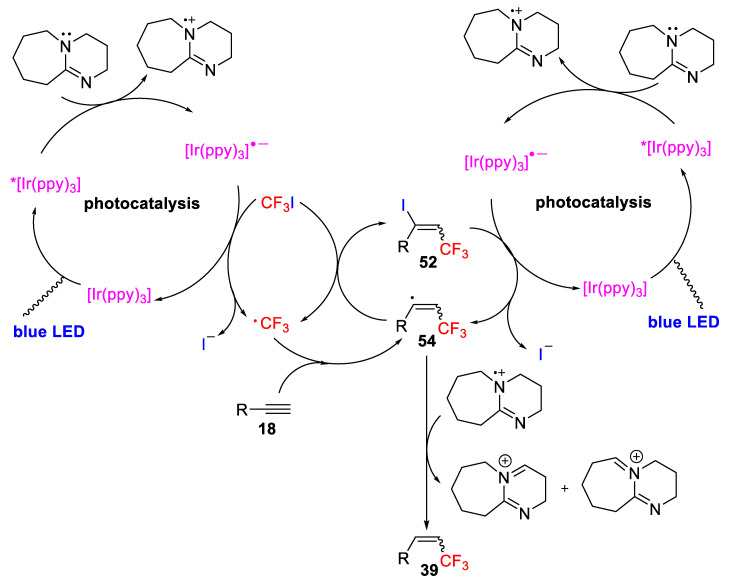
Proposed mechanism for the formation of trifluoromethylated alkenes under blue-light irradiation with iridium photocatalysts.

The authors proposed a plausible mechanism for the hydrotrifluoromethylation of alkynes in which visible light photoexcitation of [Ir(ppy)_3_] affords the excited species*[Ir(ppy)_3_], which is then reductively quenched by 1,8-diazabicyclo(5.4.0)undec-7-ene (DBU) to produce the anionic [Ir(ppy)_3_]^−^^•^ radical and the DBU radical cation. Next, the radical iridium anion performs a single-electron reduction of the F_3_C−I bond, regenerating the photocatalytic cycle and delivering a CF_3_ radical. Subsequently, addition of this radical to the alkyne produces the vinyl radical **54**, which through a direct hydrogen abstraction by the vinyl radical generates the desired alkenyl-CF_3_. Alternatively, the alkenyl–CF_3_ product could also be followed by competitive iodide abstraction from CF_3_I by the vinyl radical to give the alkenyl iodide as an intermediate, which supports the iodotrifluoromethylation of alkynes. The iodotrifluoromethylation is followed by de-iodination of the trifluoromethylated alkenyl iodide intermediate which is coupled to the same photocatalytic system as confirmed by an experimental transformation of the alkenyl iodide into the desired alkenyl–CF_3_ under the conditions for the hydrotrifluoromethylation (Scheme 43).

On the other hand, for the hydrotrifluoromethylation reaction, iridium catalysts displayed more effective activity than ruthenium catalysts with superior activity for *fac*-[Ir(ppy)_3_]. The choice of base was found to be critical, as the base also acts as a hydrogen donor. Wide scope of terminal alkynes (10 examples) underwent the hydrotrifluoromethylation under visible-light irradiation (blue LEDs) with DBU in MeCN (1:1) yielding a mixture of the *E* and *Z* hydrotrifluoromethylatedcompounds in good to excellent yields. Interestingly, *fac*-[Ir(ppy)_3_] also catalyzed the trifluoromethylation reaction of aromatic alkynes but showed to be inefficient in the trifluoromethylation of aliphatic alkynes. Aromatic alkynes were converted into trifluoromethylated alkynes with CH_3_I and *fac*-[Ir(ppy)_3_] under visible light irradiation (blue LEDs) using such bases as ^t^BuOK and Cs_2_CO_3_, but with lower yields (60–68%) than those for the iodo- and hydrotrifluoromethylation reactions. The reactions also afforded the bis(trifluoromethylated) products in 10–20% yield (Scheme 42).

As an alternative to visible-light-induced reactions which require the use of expensive ruthenium, iridium, and copper photocatalysts, organic dyes as photocatalysts have gained significant interest due to the less toxic and more sustainable properties [67,68,69]. In the last decade, several families of organic dyes, such as flavins, acridiniums, cyanoarenes, xanthenes, diaryl ketones, thiazines, among others have been exploited in the field of organic photocatalysis [70].

**Scheme 44:**
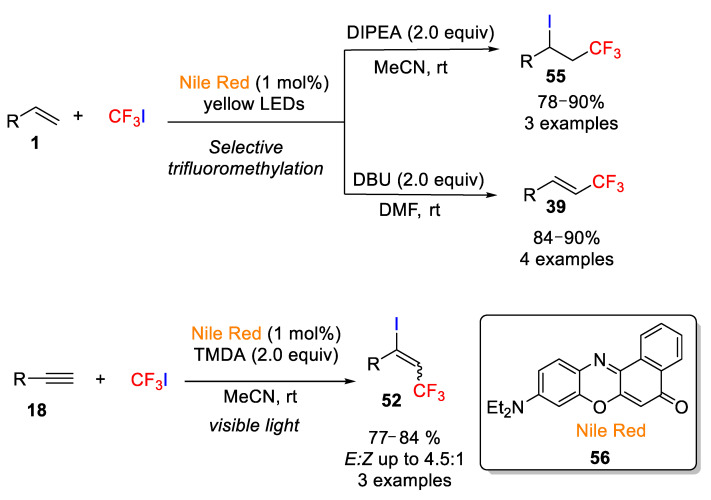
Selective trifluoromethylation of alkenes catalyzed by Nile Red under visible light irradiation.

In contrast to the widely used transition-metal complexes as photosensitizers in the iodotrifluoromethylation reactions, only a few examples have been described using organic sensitizers as catalyst in this kind of organic transformations. One of the rare examples in this field was described in 2017 by Cho’s group, who reported the trifluoromethylation of alkenes and alkynes with trifluoroiodomethane (CF_3_I) as fluorine source in the presence of the inexpensive organic dye Nile Red (**56**, 9-diethylamino-5H-benzo[α]phenoxazine-5-one) [71]. In this light-assisted process, the selective transformation of alkenes into either alkenyl-CF_3_ compounds or trifluoromethylated alkyl iodides **55** was achieved depending on the reaction conditions, in particular, the appropriate selection of the base and the solvent, which notably affected the selectivity of the process (Scheme 44). After assaying different organic dyes, bases, and solvents, the authors found that the environmentally benign organic dye Nile Red was very efficient as a photosensitizer and it was used for the first time in organic transformations. Furthermore, *N*,*N*-diisopropylethylamine (DIPEA) as base in acetonitrile was shown to be the most appropriate solvent in the iodotrifluoromethylation of alkenes, while DBU and DMF were the optimal base and solvent in the synthesis of alkenyl-CF_3_ derivatives, respectively. The scope was also studied and under the optimized conditions, aliphatic alkenes with a wide range of functional groups were easily converted into the target molecules with good chemical yields. Interestingly, the process was not appropriate for aromatic alkenes. This study was also extended to alkynes with similar results but in this case, 2 equiv of *N*,*N*,*N*’,*N*’-tetramethylethylenediamine (TMEDA) as base in MeCN were required under either yellow LED or with a compact fluorescent lamp (CFL) irradiation (Scheme 44). Additionally, this protocol was also efficient in the trifluoromethylation of some *N*-heterocycles, such as *N*-methyl pyrrole and 3-methylindole.

**Scheme 45:**
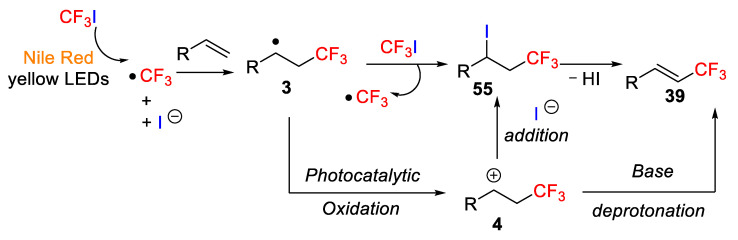
Proposed mechanism for the selective trifluoromethylation of alkenes.

A quite simple mechanism was proposed for the above transformations. Thus, a CF_3_ radical was generated by photocatalysis with Nile Red, which rapidly reacts with the alkene moiety to form a trifluoromethylated radical species **3**, followed by an atom transfer radical addition between this new radical and CF_3_I, which allows obtaining the target molecule **55**. This, after elimination of HI by the action of the base produces the alkenyl-CF_3_ derivative. Alternatively, the final iodotrifluoromethylated derivative can be obtained from a carbocation intermediate **4** generated by photocatalytic oxidation of the initial radical (Scheme 45).

**Scheme 46:**
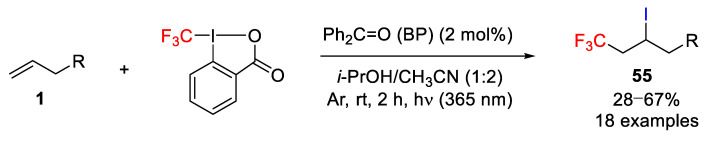
Light-promoted iodotrifluoromethylation of alkenes with benzophenone as a photosensitizer.

Vincent and co-workers studied, for the first time, the use of Togni reagent as the source of CF_3_ and iodine and benzophenone as a photosensitizer for the light-promoted iodotrifluoromethylation of alkenes (Scheme 46) [72,73]. They observed a fast reaction of the benzophenone triplet-state (^3^BP) with isopropanol to generate an α-hydroxyisopropyl radical, which could be used to reduce the Togni reagent to afford a reactive trifluoromethyl radical, 2-iodo benzoic acid, and acetone. Several control experiments were carried out to show that both *i*-PrOH and light were crucial to obtaining the best yields and that benzophenone notably increased the reaction rate. Concerning the scope (18 examples), the process worked well with a wide range of olefins although the chemical yields were only moderate. In some cases, however, the process failed particularly with styrene, and electron-deficient alkenes (i.e., phenyl vinyl sulfone and others), for which unexpected hydrotrifluoromethylated products were almost exclusively obtained.

**Scheme 47:**
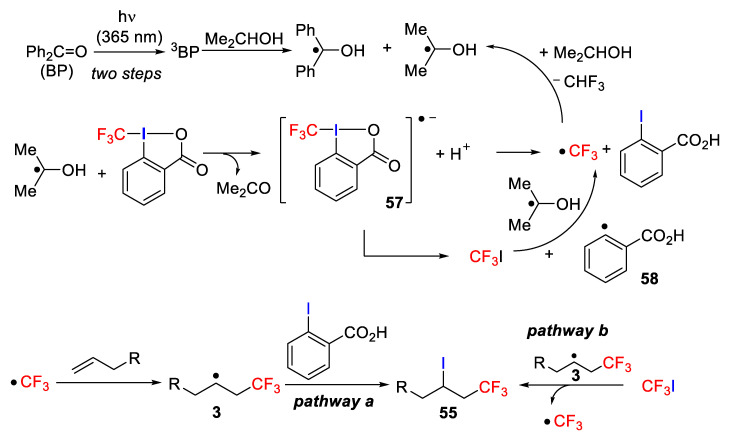
Proposed mechanism for the light-promoted iodotrifluoromethylation of alkenes with benzophenone as photosensitizer.

The authors proposed a quite sophisticated mechanism, in which an α-hydroxyisopropyl radical was initially generated by the reaction of isopropanol with BP triplet state (^3^BP). Next, α-hydroxyisopropyl radical reacted with the Togni reagent to produce CF_3_ radicals and 2-iodobenzoic acid through the anion-radical intermediate **57**. The CF_3_ radicals react very quickly with *i*-PrOH to yield CF_3_H and regenerate α-hydroxyisopropyl radical, thus maintaining the radical chain cycle. Additionally, through the decomposition of intermediate **66**, trifluoroiodomethane (CF_3_I) was formed along with benzoic acid radical **58**, which later reacts with *i*-PrOH to produce benzoic acid regenerating α-hydroxyisopropyl radical. The CF_3_ radical adds, next, to the olefin achieving, through of the α-CF_3_ alkyl radical **3**, the target molecule after final abstraction of an iodine atom from 2-iodobenzoic acid (pathway a). Alternatively, the CF_3_I generated also in situ from intermediate **57** can also react with radical **3** to obtain the final iodotrifluoromethylation molecule **55** and regenerate the CF_3_ radical (pathway b) (Scheme 47).

**Scheme 48:**
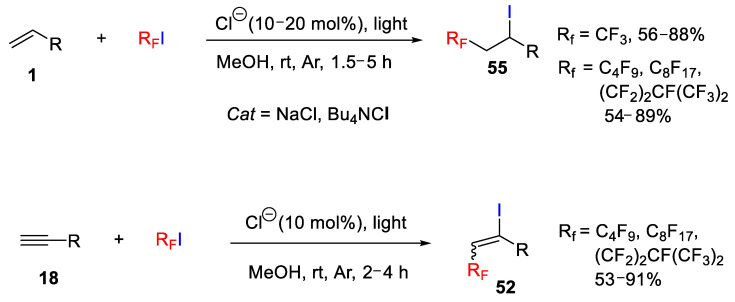
Light-mediated iodoperfluoroalkylation of alkenes/alkynes catalyzed by chloride ions.

One year later, the same group reported [74] that catalytic amounts of chloride ions (i.e., NaCl or Bu_4_NCl) could promote the iodotrifluoromethylation and iodoperfluoroalkylation of alkenes and alkynes under low intensity UVA irradiation in deoxygenated methanol solutions (Scheme 48). Additionally, in another paper by Matile and coworkers [75], the authors demonstrated that chloride ions can be transported through membranes using perfluoroalky iodides (R_F_I) as transporters and, therefore, the chloride ions might be used to weaken the R_f_-I bond through destabilization, thus facilitating the homolytic cleavage of the R_f_-I bond by the halogen bonding interaction between R_f_I and the chloride ions under direct photolysis. It is noteworthy that the direct photolysis of R_f_I using a conventional TLC lamp was shown to be inefficient (<5% yield), but the addition of a stoichiometric amount of Bu_4_NCl allowed the isolation of the desired compound in high yield (>95%). The optimized reaction conditions require the use of less than 10 mol% of the catalyst in a short irradiation time (2–4 h) and the use of methanol or ethanol as solvents. Interestingly, among all the halogens tested in the study, chloride ions were shown to be the most efficient (chloride salts such as NaCl and Bu_4_NCl exhibited similar catalytic activity) but in the case of the bromide and iodide ions, no reaction occurred. Fluorine ions, on the other hand, provided only a modest efficiency (32% yield). The scope of the process was also studied, and found that linear alkenes with different functional groups and internal alkenes were efficient and the final products were obtained in good (up to 89%) chemical yields (Scheme 48). This protocol was also successfully extended to terminal alkynes and good chemical yields were obtained in a short reaction time but with low to moderate stereoselectivity. However, CF_3_I was found to be unreactive towards alkynes.

**Scheme 49:**
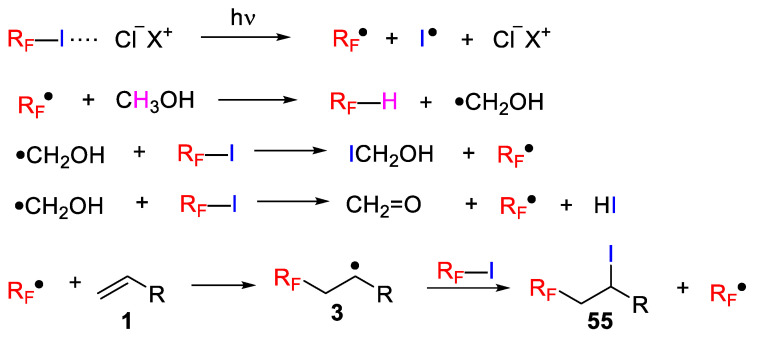
Proposed mechanism for the light-mediated iodoperfluoroalkylation of alkenes/alkynes catalyzed by chloride ions.

The authors proposed a plausible mechanism based on experimental observations: the catalytic amount of the chloride ions has a strong impact on the production of the perfluoroalkyl radicals (R_F_^.^) when the experiment is carried out in absence of alkenes (Scheme 49). Thus, the initially perfluoroalkyl radical generated from R_F_I and Cl^−^ ions and light irradiation would initiate a radical-chain reaction, which reacts rapidly with methanol to generate R_F_H and an α-hydroxymethyl radical. Then, this newly formed radical reacts with R_F_I to produce either iodomethanol or formaldehyde to regenerate the initial perfluoroalkyl radical and/or HI reinitiating the catalytic cycle. Both iodomethanol and/or formaldehyde are finally converted into formaldehyde methyl acetal by reaction with methanol. In the presence of an alkene, the formation of R_F_H was not observed, indicating a faster rate of addition of R_F_^.^ on the double bond than the hydrogen atom abstraction of methanol.

**Scheme 50:**
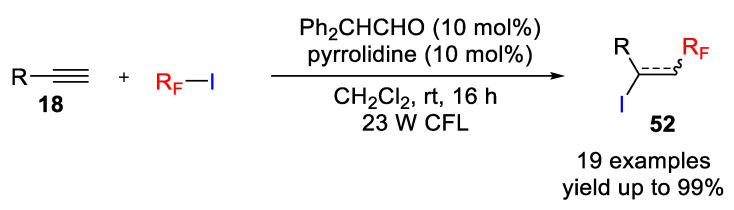
Visible light-induced radical iodoperfluoroalkylation of akenes catalyzed by enamines and amines.

Interestingly, iodoperfluoroalkylation of unactivated olefins and alkynes can also be accomplished by photo-induced-ATRA of perfluoroalkyl iodide using simple organic compounds, such as enamines and amines as catalysts. In a recent publication, Yajima and coworkers explored the photo-assisted-ATRA reaction of alkenes and alkynes with perfluoroalkyl iodides catalyzed by an in situ generated enamine species and free amine (Scheme 50) [76]. In this regard, the authors found that the combination of diphenylacetaldehyde with pyrrolidine (10 mol%) efficiently catalyzed the iodoperfluoroalkylation reaction under visible light irradiation using a 23 W fluorescent lamp. Different light sources were also investigated, and the use of a green LED decreased the yield to 89%; whereas blue or white LEDs led to excellent yields and no reaction took place in the dark. Under the optimized conditions, the scope of a variety of perfluoroalkyl iodides and olefins/alkynes was explored and a high functional group tolerance was achieved. Interestingly, reaction with preformed enamine in the absence of a free amine did not proceed efficiently but the addition of a free amine improved the yield suggesting the role of the free amine in catalyzing the reaction.

**Scheme 51:**
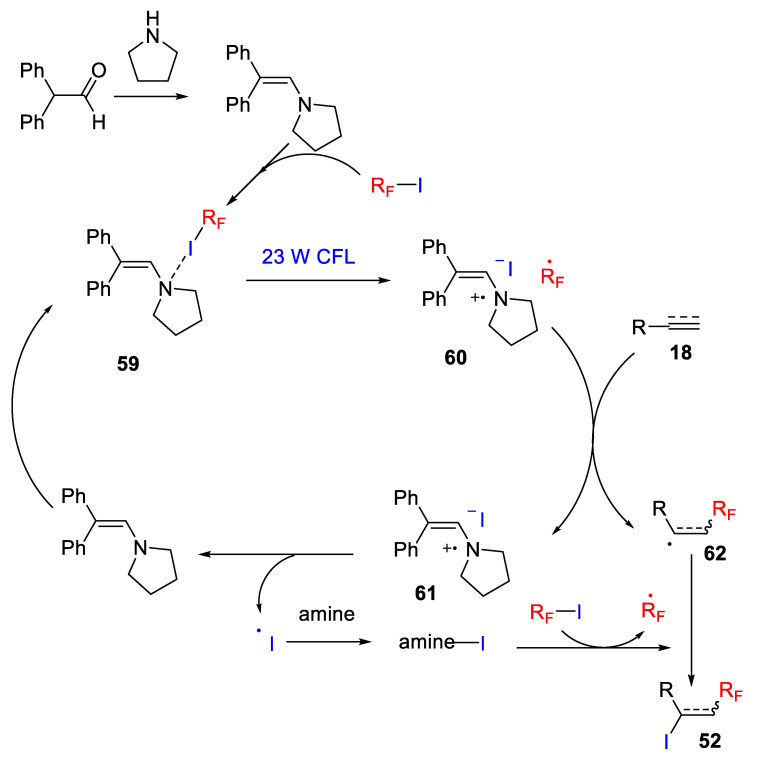
Proposed mechanism for the iodoperfluoroalkylation of alkenes catalyzed by enamines and amines.

The authors proposed a mechanism via the formation of an Electron Donor-Acceptor (EDA) complex between the perfluoroalkyl iodide and the in situ formed enamine from pyrrolidine and the aldehyde, as confirmed by optical absorption spectra. This EDA complex **59** generates a fluoroalkyl radical upon irradiation with light, which is added onto the alkene/alkyne to deliver a radical intermediate and an amine radical cation **60**. The iodoperfluoroalkylated product is formed by partial iodine transfer from another perfluoroalkyl iodide. Simultaneously, the iodide ion reacts with the enamine radical cation to produce the iodine radical, and the enamine is regenerated. The iodine radical forms a complex with the free amine and this iodide–amine complex can effectively deliver the iodine radical to radical intermediate (Scheme 51).

**Scheme 52:**
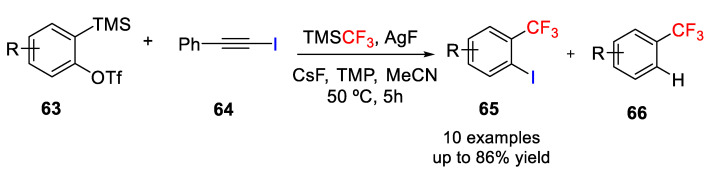
Ag-mediated vicinal trifluoromethylation−iodination of arynes.

Hu and coworkers reported an Ag-mediated vicinal trifluoromethylation−iodination protocol of arynes to afford *o*-trifluoromethyl iodoarenes **65** (Scheme 52) [77]. In this approach, trifluoromethylsilver (AgCF_3_) was prepared in situ by mixing TMSCF_3_ and AgF in 1:1 ratio and 1-iodophenylacetylene (**64**) was added as electrophile of benzyne to promote the iodination of benzyne. The optimized reaction conditions also need an external additive/ligand such as the hindered secondary amine 2,2,6,6-tetramethylpiperidine (TMP), to accelerate the iodination process and favor the formation of the iodinated product over the protonated one. The scope of the reaction was evaluated through a variety of structurally diverse aryne precursors bearing functional groups such as acetal, bromo, and allyl. Furthermore, high regioselectivity was observed in the reaction with 3-substituted benzynes as a single isomer was formed; however, 4-substituted benzynes gave almost 1:1 ratio of regioisomers.

**Scheme 53:**
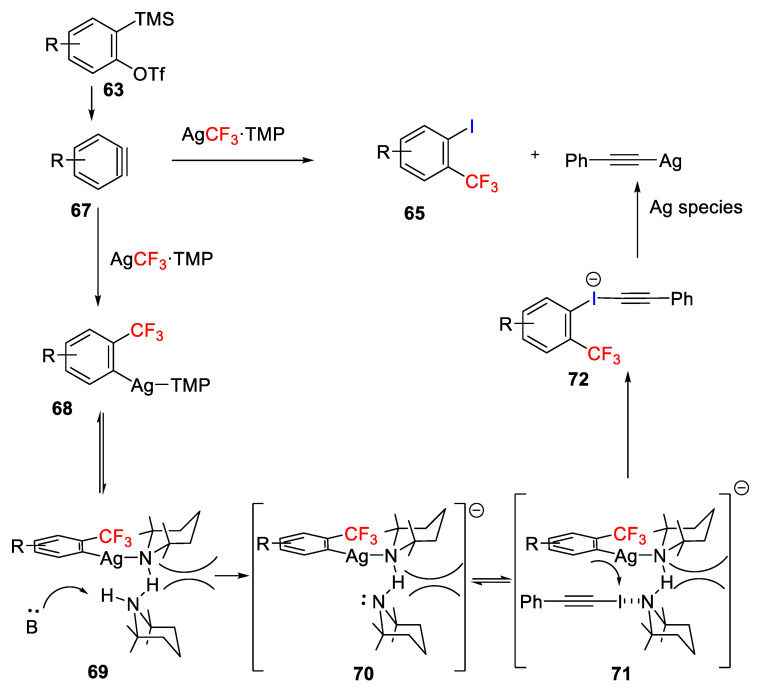
Proposed mechanism for Ag-mediated iodotrifluoromethylation of arynes.

The in situ-generated aryne **67** reacts with the formed AgCF_3_·TMP via a carboargentation reaction to give intermediate **68**, which can afford intermediate **69** via a hydrogen bond formation with another TMP, which after deprotonation generates anionic intermediate **70**. Then, iodophenylacetylene can coordinate to intermediate **80** via halogen bonding forming intermediate **81**, which undergoes nucleophilic attack to form intermediate **72**, which readily affords the desired substituted *o*-iodotrifluoromethylbenzene and silver phenylacetylide as a byproduct (Scheme 53).

**Scheme 54:**
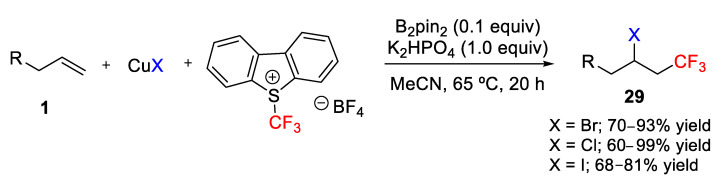
Copper(I)-mediated halotrifluoromethylation of alkenes.

A copper-mediated halotrifluoromethylation of alkenes was reported by Jung, Han, and coworkers in 2015 using Umemoto reagent and a copper(I) halide salt, which would form a transition metal halide that generates the active CF_3_ species and at the same time act as the halogen source (Scheme 54) [78]. The main advantage of this approach is the high stability, commercial availability, and relatively low cost of copper halides. With this approach, bromotrifluoromethylation, chlorotrifluoromethylation, and iodotrifluoromethylation of terminal alkenes was achieved with moderate to high yields (60–99%) simply by selecting the copper(I) halide, CuBr, CuCl, and CuI, respectively. The use of bis(pinacolato)diboron (B_2_pin_2_) as an additive was found to be very efficient in terms of increasing the yield of the β-halotrifluoromethylated product. It is noteworthy that iodotrifluoromethylation using CuI and Umemoto reagent stands as an alternative to the problematic use of gaseous CF_3_I. Finally, 1,1-disubstituted and internal alkenes were also converted into the corresponding halotrifluoromethylated products in high yields, tolerating the presence of other functional groups such as α,β-unsaturated aldehydes and ketones.

**Scheme 55:**
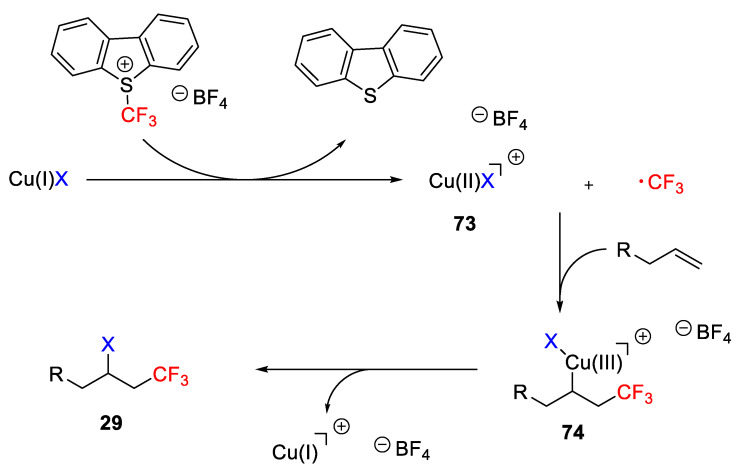
Proposed reaction mechanism for the copper-mediated halotrifluoromethylation of alkenes.

The authors proposed the mechanistic pathway displayed in Scheme 55. Initially, the copper halide activates the CF_3_ source upon reaction with Umemoto reagent by oxidation of Cu(I) to the Cu(II) species (**73**) and generating the CF_3_ radical. This CF_3_ radical reacts with the alkene to give an alkyl radical, which is recombined with the previously generated Cu(II) species to afford the Cu(III) species (**74**). Finally, reductive elimination delivers the desired halotrifluoromethylated product (Scheme 55).

**Scheme 56:**
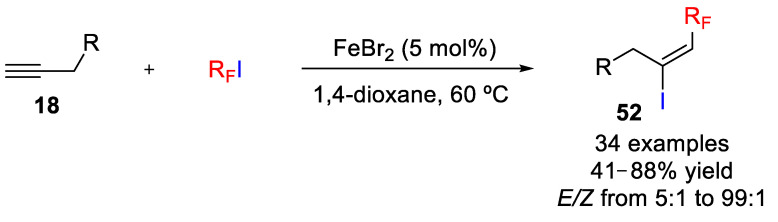
Iron-catalyzed 1,2-addition of perfluoroalkyl iodides to alkynes.

Hu and coworkers reported an iron-catalyzed 1,2-addition of perfluoroalkyl iodides (R_F_I) to alkynes with a wide substrate scope (up to 34 examples) and high functional-group tolerance (Scheme 56) [79]. This iron-based methodology is based on the use of FeBr_2_ at low loadings (5 mol%) and Cs_2_CO_3_ (0.8 equiv) as base, despite other transition metal catalysts such as CuI, CoBr_2_, Cu(OTf)_2_, or NiCl_2_·dme, were also effective but in lower yields. The role of the base and running the reaction under a nitrogen atmosphere were found to be critical, the reaction did not occur either without a base or under air. The use of Et_3_N as an alternative base gave modest yields but no conversion was observed when using other bases such as *t*BuOLi, *t*BuOK, and pyridine. Interestingly, this iodoperfluorination reaction also occurred with only Cs_2_CO_3_ as the base and without the iron catalyst, but reaction conditions required a high concentration of reagents and lowering the base loading to 0.25 equivalents. Under the optimized conditions, alkyne (0.5 mmol), R_F_I (1.5 equiv), FeBr_2_ (10 mol%), Cs_2_CO_3_ (0.8 mmol), in 1,4-dioxane at 60 °C, a great number of substrates with diverse functional groups were efficiently iodoperfluorinated using C_4_F_9_I, C_6_F_13_I, but also C_6_F_12_ClI and EtOOCCF_2_I. The addition was found to be *E* selective, with an *E/Z* ratio higher than 5:1, and completely stereoselective with aryl alkynes, which yielded only *E* isomers. The authors demonstrated that the reaction mechanism is consistent with the formation of a perfluoroalkyl radical and an iodide radical by reaction of the perfluororoalkyl iodide with the base and its subsequent addition to the unsaturated C-C, which is activated by the iron catalyst to form an alkenyl radical. This radical reacts with the previously generated iodide radical to deliver the iodoperfluorinated product. The iron-catalyzed iodoperfluorination was also expanded to both terminal and internal alkenes with a wide tolerance of functionalities (ester, keto, aryl-halide, and ether groups), which delivered the iodoperfluorinated product in moderate to high yields (64–95%) and in the case of internal olefins, stereoisomers were obtained in a 1:1 diastereomeric ratio.

**Scheme 57:**
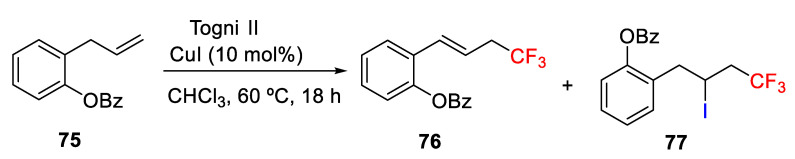
Copper-catalyzed halotrifluoromethylation with substrates containing allylic C-H bond.

In addition, Cu-catalyzed halotrifluoromethylation reactions of substrates with allylic C-H bonds using Togni reagent were reported by Szabó and co-workers [80]. In this work, iodotrifluoromethylation with a stoichiometric amount of CuI was accomplished, affording allylic trifluoromethylated **76** and addition products **77** in moderate yields (Scheme 57). Szabó’s group also reported a copper-mediated cyanotrifluoromethylation of styrenes using the Togni reagent as the CF_3_ source in the presence of catalytic amounts of PCy_3_ [81]. However, iodotrifluoromethylation was afforded when using CuI instead of the CuCN employed for the cyanotrifluoromethylation reaction.

**Scheme 58:**
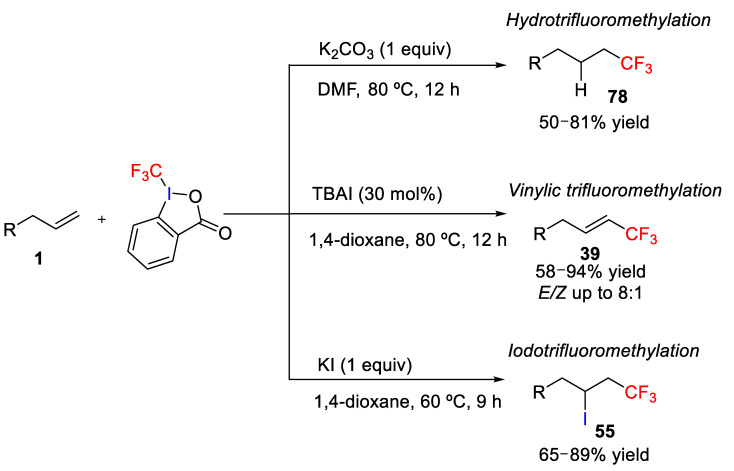
Product control in alkene trifluoromethylation of alkenes using Togni reagent.

Interestingly, a product control in alkene trifluoromethylation using Togni reagent was reported by Sodeoka and coworkers [82]. In this work, the hydrotrifluoromethylation, vinylic trifluoromethylation, or iodotrifluoromethylation product could be selected by tuning the reaction conditions (Scheme 58). On one hand, the desired hydrotrifluoromethylation product **78** was achieved by using Togni reagent in the absence of any transition metal catalyst using K_2_CO_3_ as a base and DMF as solvent. Under these conditions, hydrotrifluoromethylation of several alkenes was accomplished in the presence of various functional groups, such as carbonyl groups, ester, and aryl halides, affording the corresponding products in good to moderate yields and high selectivity. Studies with deuterated solvent allowed to establish that the hydrogen source of the hydrotrifluoromethylation was the solvent, i.e., DMF. On the other hand, the vinylic trifluoromethylation product was formed with high selectivity using Togni reagent and tetra-*n*-butylammonium iodide (TBAI) in 1,4-dioxane with the *E/Z* ratio of the product up to 8:1. As also observed in the hydrotrifluoromethylation, various functional groups were tolerated in the vinylic trifluoromethylation. However, *E/Z* ratio was found to be low (2:1 ratio) in substituted alkenes. Finally, the iodotrifluoromethylation product **55** was obtained in good to high yields under the same reaction conditions for the vinylic trifluoromethylation but simply replacing of TBAI by KI. Under the optimized conditions, iodotrifluoromethylation also tolerated several functional groups; however, internal alkenes were unsuitable for the iodotrifluoromethylation due to generate of regio- and stereoisomers.

**Scheme 59:**
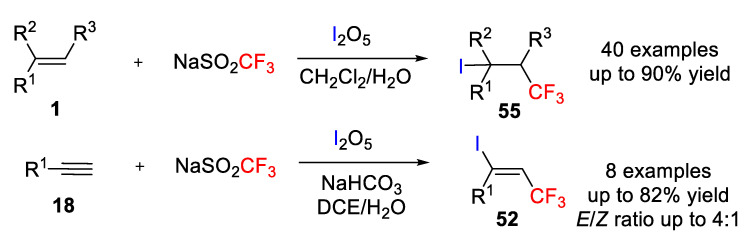
Iodotrifluoromethylation of alkenes and alkynes with sodium trifluoromethanesulfinate and iodine pentoxide.

Iodotrifluoromethylation of alkenes and alkynes can also be achieved via single electron oxidation of a CF_3_-derived anion by an inorganic hypervalent iodine oxidant. In a work by Liu and coworkers, oxidation of sodium trifluoromethanesulfinate (NaSO_2_CF_3_) generated the CF_3_ radical, which added to the unsaturated bonds, followed by the capture of the reducing substance iodine to form β-CF_3_ iodides (Scheme 59) [59]. Among several inorganic iodine sources, iodine pentoxide (I_2_O_5_) was found to be more efficient than other organic hypervalent iodine compounds such iodic acid (HIO_3_). Regarding the solvent, a 1:1 mixture of CH_2_Cl_2_/H_2_O was shown to be the best choice. Under the optimized conditions, iodotrifluoromethylation of a wide variety of alkenes and alkynes was carried out by using stable and inexpensive solid reagents CF_3_SO_2_Na and I_2_O_5_ in aqueous medium, instead of using the typical and hard to handle CF_3_I gas. A wide tolerance of functional groups (halogens, amide, ether, carbonyl, carboxylate, hydroxyl, nitro, sulfonate, and sulfamide) was also observed. In this regard, aryl- and alkyl-substituted alkenes afforded β-CF_3_ alkyl iodides in moderate to high yields (40 examples, up to 90% yield). Terminal and nonterminal alkenes show also good compatibility, but when both double bonds are present in one substrate, the iodotrifluoromethylation selectively occurred at the terminal position. Electron spin resonance (ESR) studies allowed confirming that the reaction occurred via a free-radical process through characterization of two key intermediates such as CF_3_ and the β-CF_3_ alkyl radicals, which were trapped using 2-methyl-2-nitrosopropane (MNP) as a radical spin trap. Finally, the iodotrifluoromethylation of alkynes was also accomplished by this methodology affording €-β-CF_3_ alkenyl iodides **52** with *E*/*Z* ratios up to 4:1 but with the assistance of NaHCO_3_ and a mixture of 4:1 of DCE/H_2_O as solvent.

**Scheme 60:**
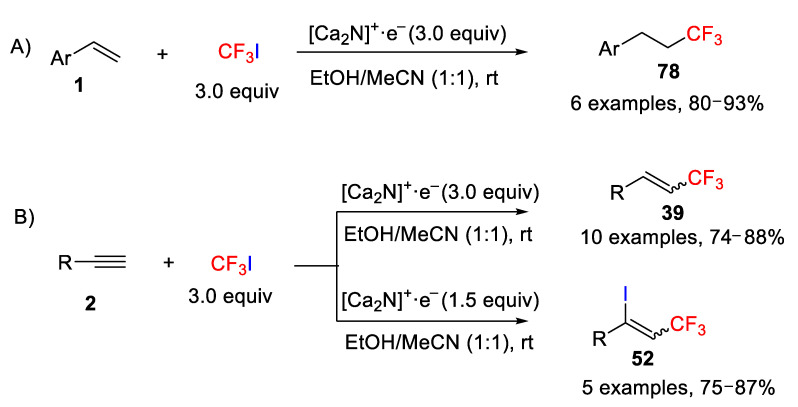
Iodotrifluoromethylation of alkenes and alkynes with CF_3_I in the presence of inorganic electrides.

The groups of Yang, Kim, and Cho reported in 2014 an interesting and environmentally benign methodology for the hydrotrifluoromethylation and iodotrifluoromethylation of unactivated alkenes and alkynes with CF_3_I in the presence of inorganic electrides (ionic salts with electrons as the anions) [83], such as polycrystalline [Ca_2_N]^+^∙e^−^, which were used as an electron source through a SET pathway [84]. The authors indicated that the solvent plays a crucial role in the method. After checking different solvents, they found that alcoholic solvents such as ethanol and acetonitrile (1:1) were the mixture of choice to carry out the reaction successfully. Initially, they studied the behavior of alkenes under the optimized reaction conditions and found that the process worked well but only with aromatic derivatives providing exclusively the hydrotrifluoromethylation products (Scheme 60A). With aliphatic alkenes, an inseparable mixture of iodotrifluoromethylated **52** and hydrotrifluoromethylated compounds **78** was obtained. However, the situation for the alkynes was different. In this case, the authors observed that under the optimized conditions, the process worked also well, for both aromatic and aliphatic alkynes, providing only the hydrotrifluoromethylation product when using 3.0 equiv of the electride [Ca_2_N]^+^∙e^−^. On the other hand, by controlling the electride amount, i.e., using only 1.5 equiv of [Ca_2_N]^+^∙e^−^, the reaction led to the selective formation of the iodotrifluoromethylation product with good yields and excellent E/Z selectivity (Scheme 60B).

**Scheme 61:**
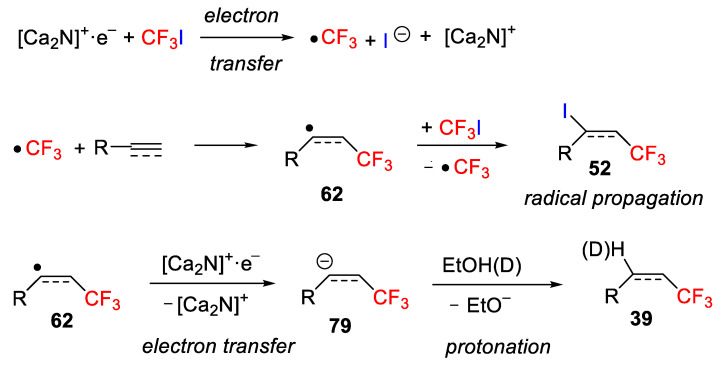
Proposed mechanism for the iodotrifluoromethylation of alkenes and alkynes with CF_3_I in the presence of inorganic electrides.

The authors proposed a tentative mechanism starting with a single electron transfer from [Ca_2_N]^+^∙e^−^ to CF_3_I to generate first a CF_3_ radical and iodide anion, which after addition to the alkene or alkyne provided a new alkyl or vinyl radical **62**. Final iodine abstraction from CF_3_I generated the target iodotrifluoromethylated product **52** and a CF_3_ radical. The hydrotrifluoromethylated products **39** were formed after a second SET from [Ca_2_N]^+^∙e^−^ to the radical intermediate **62**, followed by protonation with ethanol, which was used as an electride activator and hydrogen donor (Scheme 61).

It should be mentioned that there were also some related reports on the halo-perfluoroalkylation of unsaturated bonds which just contained one example about trifluoromethylation [84,85,86,87,88,89,90,91,92,93,94,95].

## 4. Fluorotrifluoromethylation

Fluorotrifluoromethylation reactions allow the difunctionalization of organic compounds through the introduction of CF_3_ and a fluorine atom in a single step for the construction of complex multi-functionalized trifluoromethylated derivatives with potential applications in pharmaceuticals, agrochemicals, and materials science.

**Scheme 62:**
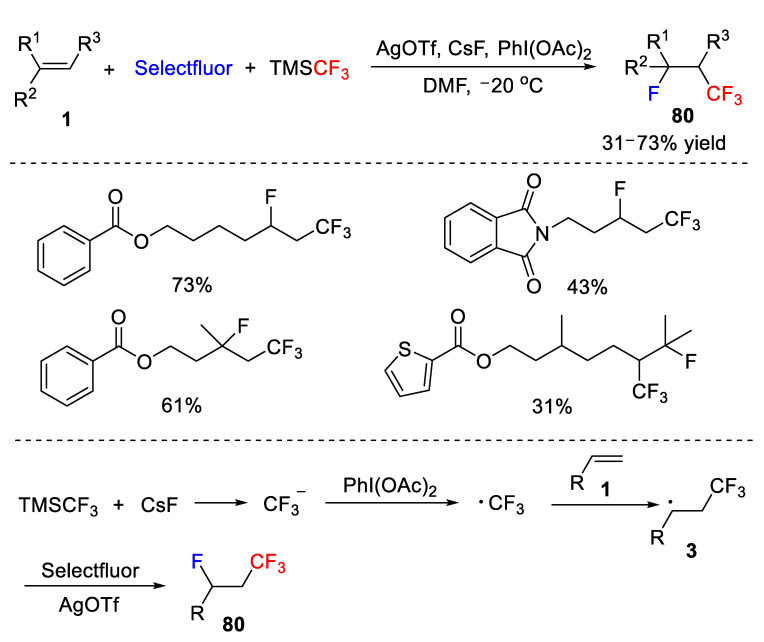
Silver promoted fluorotrifluoromethylation of alkenes.

Qing and coworkers developed an efficient difunctional fluorotrifluoromethylation of alkenes promoted by silver in 2015 (Scheme 62) [96]. Selectfluor was employed as the F source and (trifluoromethyl)trimethylsilane (TMSCF_3_) was used as the CF_3_ source. The fluorotrifluoromethylated products could be obtained in the presence of cesium fluoride (CsF), iodobenzene diacetate [PhI(OAc)_2_] and silver trifluoromethanesulfonate (AgOTf), which was proved to be very important for the reaction. Various unactivated alkenes, including disubstituted terminal alkenes and trisubstituted internal alkenes, could participate well affording the desired products **80**, along with hydrotrifluoromethylated products as major by-products in most cases. A possible reaction pathway via CF_3_ radical was presented by the authors as shown in Scheme 62. First, CF_3_ anion was generated by treating TMSCF_3_ with CsF, followed by the oxidation of PhI(OAc)_2_ leading to the CF_3_ radical. Then, CF_3_ radical added to alkene to give the trifluoromethylated alkyl radical intermediate **3**. Finally, the fluorotrifluoromethylated product **80** was obtained by AgOTf mediated fluorination using Selectfluor.

**Scheme 63:**
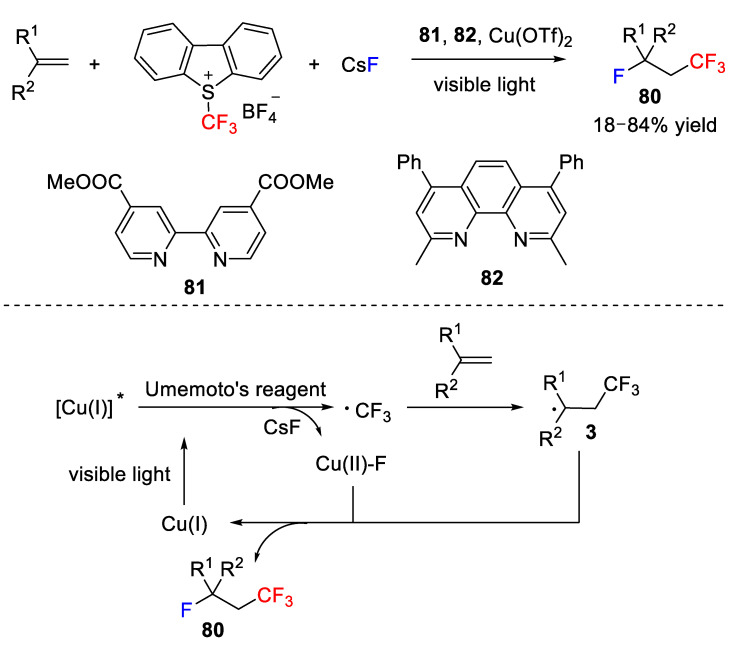
Copper-catalyzed fluorotrifluoromethylation of alkenes.

Afterward, the Yu group reported a cooper catalyzed fluorotrifluoromethylation of alkenes irradiated by visible light in 2018 (Scheme 63) [97]. Different from Qing’s work, the authors used CsF as the F source and Umemoto reagent as the CF_3_ source. The reaction tolerated a wide substrate scope of various unactivated alkenes to furnish the fluorotrifluoromethylated products **80** in moderate to good chemical yields. Importantly, bipyridine **81** and bathocuproine (**82**) were necessary ligands for this transformation along with Cu(OTf)_2_ as the catalyst. It should be mentioned that mechanistic studies strongly indicated carbocationic intermediates were not involved in the fluorination step. The final product **80** was suggested to be formed via the fluorine atom transfer directly from Cu(II)-F to alkyl radicals.

**Scheme 64:**
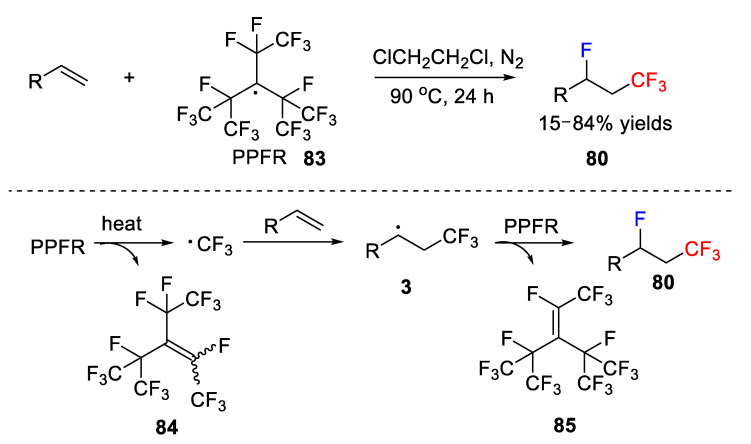
Fluorotrifluoromethylation of alkenes with perfluoroalkyl radical.

As disclosed above, although the two reported methodologies could be considered as one-step fluorotrifluoromethylation, they were based on complex reactions with the addition of more than one kind of metallic compounds. Not only the radical process but also an oxidative step was involved in the transformation. Besides, the F source and CF_3_ source usually came from two different fluorinated reagents. Against this background, Soloshonok, Ponomarenko and co-workers established an elegant mediator and additive free fluorotrifluoromethylation of alkenes in the presence of a persistent perfluoroalkyl radical (Scheme 64) [98]. Perfluoro-3-ethyl-2,4-dimethyl-3-pentyl radical (**83**, PPFR), which was extremely stable at ambient temperature in the open air, served in the reaction as both the F source and CF_3_ source. A lot of terminal alkenes could be converted into the corresponding fluorotrifluoromethylated products **80** just by heating with PPFR at 90 °C. PPFR could release a trifluoromethyl radical when heating at 90 °C, which subsequently added to alkene to generate the alkyl radical. Treatment with another PPFR, the desired difunctionalized product was achieved. Meanwhile, the proposed mechanism was proved by the detection of compounds **84** and **85** in the reaction mixture with GC technique.

**Scheme 65:**
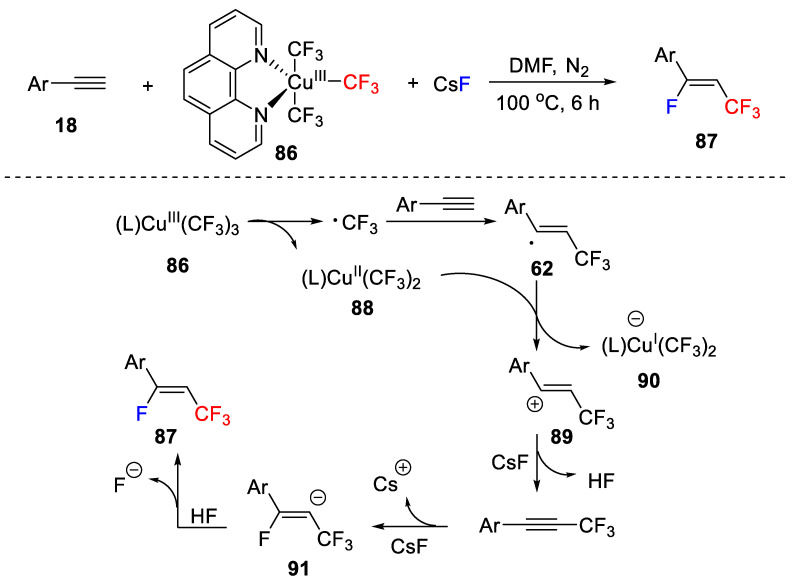
Copper mediated fluorotrifluoromethylation of terminal alkynes.

Despite several methodologies for fluorotrifluoromethylation of alkenes being developed, similar difunctionalizaiton of alkynes to access fluorotrifluoromethylated alkenes was also a great challenge. In 2017, the Zhang group presented the first general method for *syn*-fluoro-trifluoromethylation of terminal alkynes to afford (*Z*)-fluoro-trifluoromethyl alkenes **87** with excellent selectivity (Scheme 65) [99]. (L)Cu^III^(CF_3_)_3_ **86** and CsF were used as CF_3_ and F sources, respectively. The former copper complex was thought to be the key to the success of this transformation. Based on several control experiments, a plausible mechanism involving radical species was suggested. First, CF_3_ radical was given by Cu(III) **86** and then added to the triple bond furnishing trifluoromethylated vinyl radical **62**. Via single electron transfer from radical **62** to Cu(II) **88**, vinyl cation **89** was achieved along with Cu(I) **90**. Deprotonation of intermediate **89** produced arylacetylene, followed by the fluorine anion addition from CsF at the electrophilic α position to form the *syn*-α-F-β-CF_3_ styryl anion **91**. The final step was protonation of **91** to afford the (*Z*)-fluoro-trifluoromethyl styrenes.

Later, the same group developed a similar strategy for the fluorotrifluoromethylation of alkynes by employing a tertiary amine instead of CsF under the same reaction conditions [100]. Different from their previous work, Cu(III) **86** acted as both the F and CF_3_ sources and an ammonium hydrogen bonding assisted α-F elimination of **86** was suggested to be involved in this transformation.

**Scheme 66:**
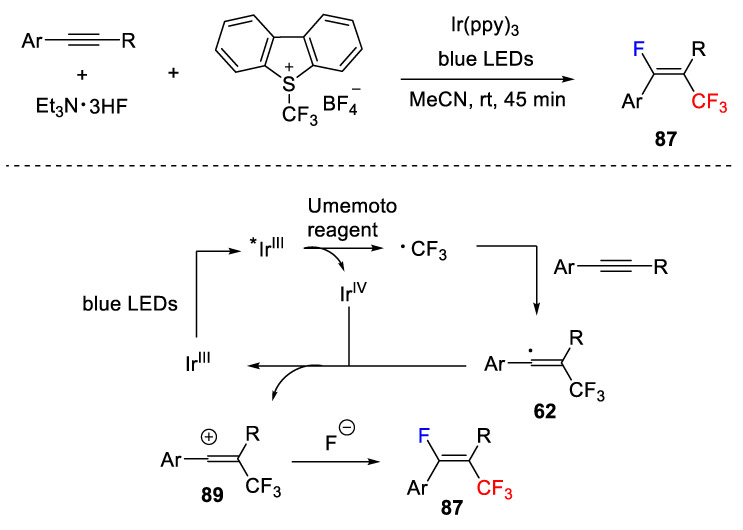
Photoredox catalyzed fluorotrifluoromethylation of internal alkynes.

Han, Kim, and coworkers reported an efficient photoredox catalyzed difunctionalization of internal alkynes for the synthesis of tetrasubstituted alkenes with fluorine and trifluoromethyl group (Scheme 66) [101]. In this procedure, Umemoto reagent remained the choice for CF_3_ source, while F came from triethylamine trihydrofluoride (Et_3_N**^.^**3HF). The reactions occurred smoothly under mild conditions in the presence of Ir(ppy)_3_ as photocatalyst, tolerating various functional groups on the alkyne skeletons. As indicated by the author, the photoredox catalytic cycle was initiated by irradiation with blue LEDs to excite Ir^III^ to *Ir^III^, which then reduced the Umemoto reagent generating CF_3_ radical along with Ir^IV^. CF_3_ radical added to alkyne forming the new vinyl radical **62**, which followed by oxidation by Ir^IV^ to give the carbocation intermediate **89**. Here, the concomitant Ir^III^ from reduction of Ir^IV^ went back into further photoredox cycles. The last step was the coupling reaction of carbocation **89** and fluorine anion from Et_3_N**^.^**3HF, allowing the desired tetrasubstituted alkenes. It was particularly noteworthy that (*E*)-fluoro-trifluoromethyl alkenes were obtained in contrast to the above methodologies.

## 5. Cyano-trifluoromethylation

The cyano group belongs to an important carbon-containing unit for synthesis because this group can be easily further converted into other functional moieties. Due to its synthetic versatility, cyanotrifluoromethylation of alkenes also represents high-valued reaction and has attracted much attention over the past years.

**Scheme 67:**
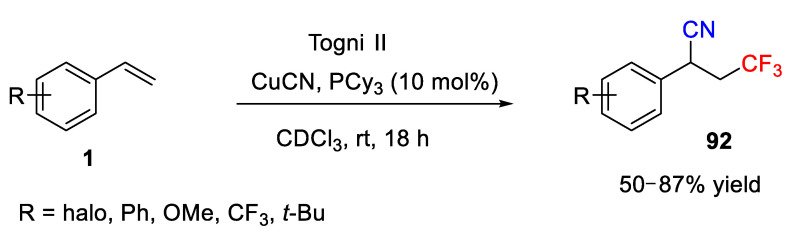
Cu-promoted cyanotrifluoromethylation of alkenes.

In 2013, Szabó and coworkers reported a Cu/phosphine-promoted cyanotrifluoromethylation of aryl alkenes with Togni II reagent as trifluoromethyl source (Scheme 67) [81]. They successfully employed CuCN as the cyano source, which smoothly reacted with a diverse set of aryl alkenes to give the corresponding difunctionalized product **92**. The reaction was carried out at room temperature and moderate to good chemical yields (50–87%) were obtained after 18 h. It should be mentioned that deuterated chloroform was used as the solvent for this reaction, as the authors investigated the crude reaction mixture with ^1^H and ^19^F NMR spectroscopy.

**Scheme 68:**
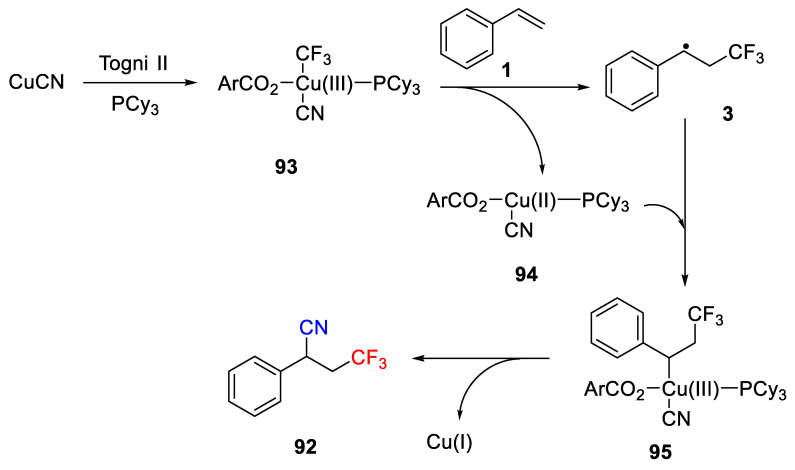
Possible mechanism for Cu-promoted cyanotrifluoromethylation of alkenes.

As shown in Scheme 68, a radical mechanism was proposed for this Cu-catalyzed cyanotrifluoromethylation reaction. Initially, CuCN is oxidized by Togni II in the presence of phosphine ligand to give the Cu(III) complex **93**, which undergoes the homolytic cleavage to generate the CF_3_ radical and followed by addition to C=C double bond. Thus, the radical intermediate **3** is obtained as well as the formation of Cu(II) complex **94**. Subsequently, the radical intermediate **3** oxidizes the Cu(II) complex **94** to form the Cu(III) complex **95**. Finally, reductive elimination of the Cu(III) complex **95** affords the desired cyanotrifluoromethylated product **92** as well as the Cu(I) complex for the next catalytic cycle.

**Scheme 69:**
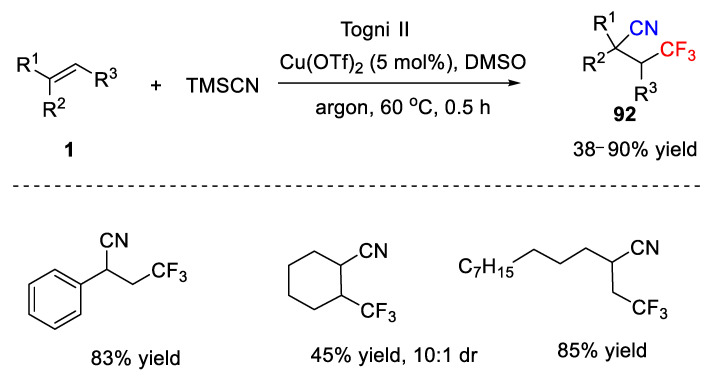
Cu-catalyzed cyanotrifluoromethylation of alkenes.

In addition to the use of CuCN as the cyano source, TMSCN has also been developed as the cyano source for the cyanotrifluoromethylation of alkenes. In 2014, Liang and co-workers reported an efficient cyanotrifluoromethylation reaction of alkenes using Togni II and TMSCN as trifluoromethyl and cyano source respectively under ligand-free and additive-free conditions (Scheme 69) [102]. Comparing with the work from the Szabó group, this difunctionalization reaction was carried out with a catalytic amount of Cu(OTf)_2_ and no phosphine ligand was needed for the conversion. Furthermore, this reaction features a good functional group tolerance and mild conditions, affording a variety of useful CF_3_-containing nitriles in 38–90% yield. Styrene worked well in this reaction and generated the desired product **92** in 83% yield. In addition, an alkyl alkene, such as cyclohexene, was a suitable substrate for this system with moderate yield and good diastereoselectivity (10:1 dr). Additionally, the authors also performed further synthetic transformation of the obtained CF_3_-containing nitriles, and the corresponding CF_3_-containing amine and carboxylic acid were obtained in good yields.

**Scheme 70:**
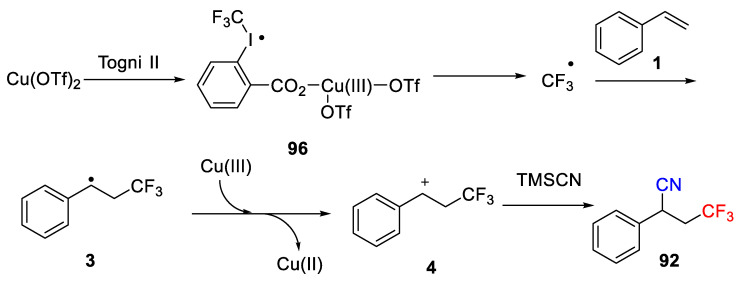
Possible mechanism for Cu-catalyzed reaction.

A possible mechanism for this Cu-catalyzed reaction with TMSCN as cyano source was postulated by the authors as shown in Scheme 70, which is similar with the previous report. First, Togni reagent oxides Cu(II) catalyst to generate Cu(III) complex **96**, which releases a trifluoromethyl radical. Then, addition of trifluoromethyl radical to C-C double bond gives the benzylic radical intermediate **3**. Subsequently, the radical **3** is oxidized by Cu(III) to give cation **4**, which then directly couple with TMSCN to form the desired product **92**.

**Scheme 71:**
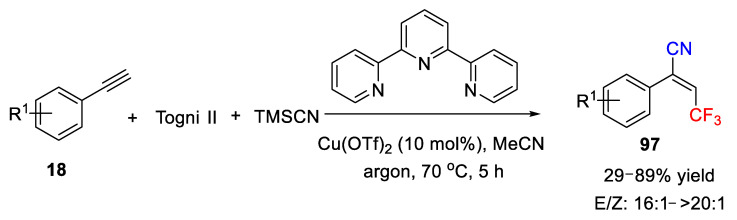
Cu-catalyzed cyanotrifluoromethylation of alkynes.

Afterward, the same group extended this Cu-catalyzed intermolecular cyanotrifluoromethylation reaction to alkynes (Scheme 71) [103]. This Cu(OAc)_2_-catalyzed reaction was carried out under similar reaction conditions, and employed TMSCN and Togni reagent II as cyano and trifluoromethyl precursor, respectively. However, addition of 2,2’:6’,2’’-terpyridine ligand was essential for the transformation. Aryl alkynes were suitable substrates and good yields were obtained. On the sharp contrary, this reaction outcome was sensitive to the substitution on the C-C triple bond. A dramatically lower yield (30%) was found when the C-C triple bond features an alkyl group, instead of an aryl group. This reaction also showed excellent stereoselectivity, and the ratio of E/*Z* was from 16:1 to >20:1.

In a related process, Liu and co-workers reported an alternative mild cyanotrifluoromethylation reaction of alkenes by use of 10 mol% CuBr as a catalyst with up to 98% yields in 2014 [104].

**Scheme 72:**
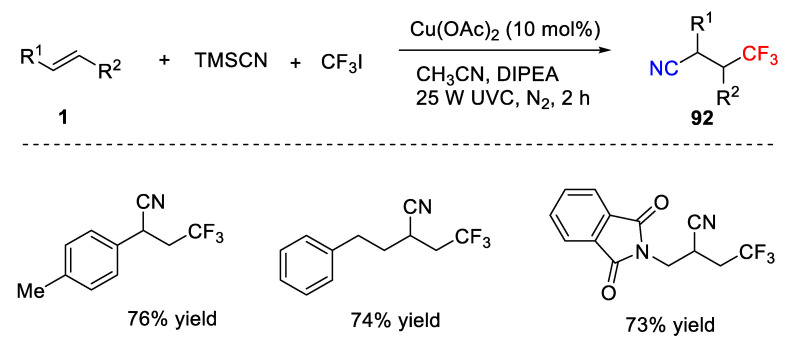
Cu-catalyzed cyanotrifluoromethylation of alkenes with fluoroalkyl iodides.

The following research interests in the area of cyanotrifluoromethylation of alkenes mainly focused on the development of new trifluoromethyl sources. Xu, Wang, and co-workers developed a cyanofluoroalkylation reaction of alkenes with fluoroalkyl iodides as fluoroalkylation sources (Scheme 72) [105]. This strategy used trifluoromethyl iodide as substrate, which could be efficiently converted into trifluoromethyl radical under photoredox conditions. Interestingly, the alkyl substituted alkenes were suitable substrates and the corresponding vicinal trifluoromethyl nitriles were obtained in good yields.

**Scheme 73:**
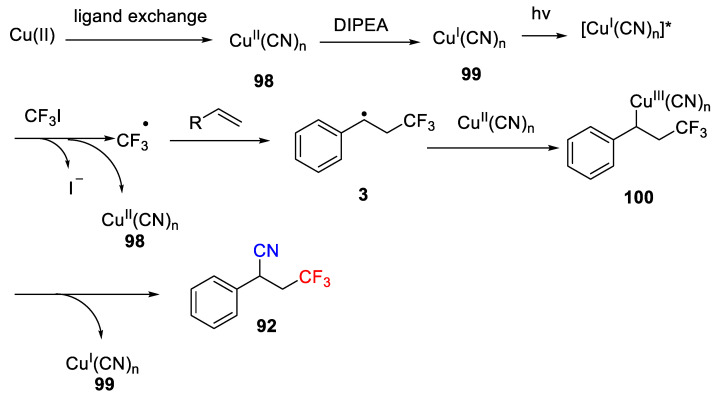
Possible mechanism for Cu-catalyzed reaction.

The authors also conducted some control experiments to get insight into the mechanism. They found that no corresponding cyanotrifluoromethylated product was formed when a radical inhibitor TEMPO was added, which indicates the putative radical mechanism. The proposed mechanism was provided in Scheme 73, which starts from ligand exchange of Cu(II) catalyst to generate Cu(II) complex **98**. Then, reduction of Cu(II) complex **98** by DIPEA happens to form the Cu(I) complex **99**, which is excited 99* under photo irradiation. Subsequently, reaction of CF_3_I with complex 99* affords trifluoromethyl radical **3** as well as Cu(II) complex **98**. Addition of trifluoromethyl radical to alkene, followed by reduction by Cu(II) complex **98** generates intermediate **100**, which undergoes reductive elimination to give the corresponding difunctionalized product **92** as well as Cu(I) complex **99** for the next catalytic cycle.

**Scheme 74:**
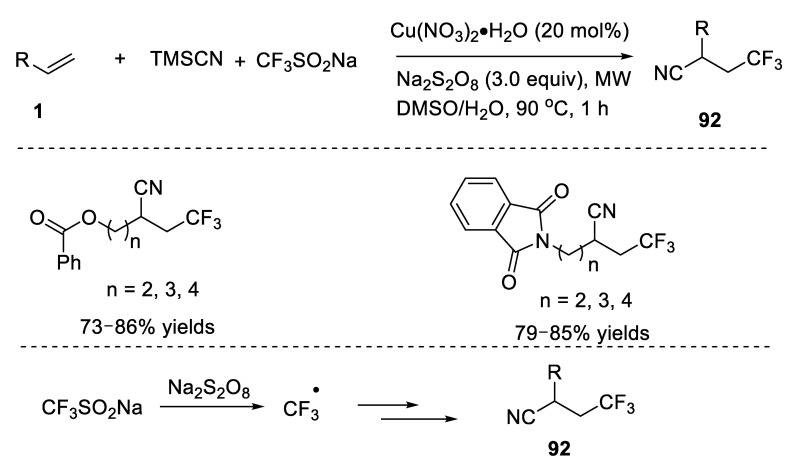
Cu-catalyzed cyanotrifluoromethylation reaction with sodium trifluoromethanesulfinate.

Then, sodium trifluoromethanesulfinate was developed as trifluoromethyl radical for the related Cu-catalyzed intermolecular cyanotrifluoromethylation of unactivated alkenes (Scheme 74) [106]. The reaction was conducted in a co-solvent (DMSO/water) with Na_2_S_2_O_8_ as an oxidant under microwave conditions. This reaction proceeded smoothly at 90 °C and afforded the corresponding product **92** in good chemical yields. In particular, unactivated alkenes were employed as substrates, which represents an alternative and efficient method for the preparation of β-trifluoromethyl nitriles.

The authors also performed control experiments with addition of TEMPO and BHT, which dramatically suppressed the reaction. Thus, the reaction proceeded through a similar process with trifluoromethyl radical as a key intermediate. The first step is the generation of trifluoromethyl radical via oxidation of sodium trifluoromethanesulfinate by Na_2_S_2_O_8_. Then, the reaction proceeds through the same oxidative addition and reductive elimination steps to form the desired product.

**Scheme 75:**
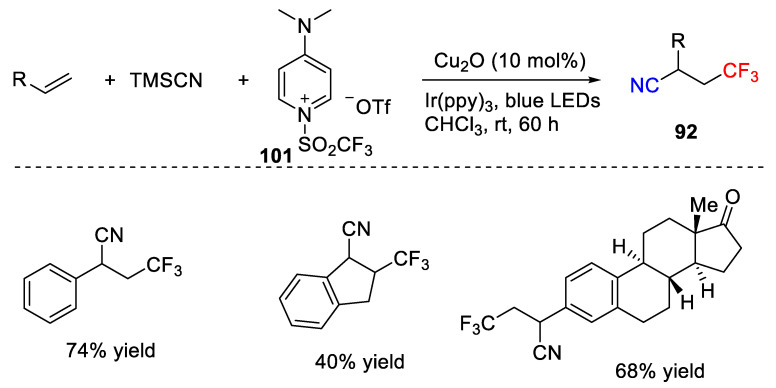
Cu-catalyzed cyanotrifluoromethylation reaction of alkenes.

Trifluoromethylsulfonyl−pyridinium salt (**101**, TFSP) is an air stable reagent, which can be easily prepared from the reaction of Tf_2_O with 4-(dimethylamino)-pyridine. Very recently, Xiao and co-workers employed this TFSP reagent as a trifluoromethyl radical precursor and successfully applied it in the cyanotrifluoromethylation difuctionalization reaction of alkenes (Scheme 75) [107]. This reaction was conducted in the presence of blue LEDs with Cu_2_O and Ir(ppy)_3_ as co-catalyst, affording the corresponding trifluoromethylated nitriles **92** in moderate to good chemical yields. It should be mentioned that only styrene and its derivatives have been used as alkenes substrates, in particular, an estrone derivative also was a suitable substrate for this reaction.

**Scheme 76:**
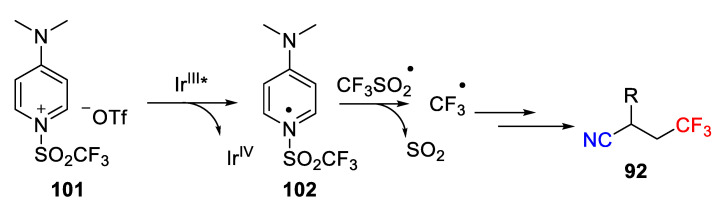
Possible mechanism for Cu-catalyzed cyanotrifluoromethylation reaction.

The proposed mechanism for this photo-promoted difunctionalization reaction with TSFP as a radical source is presented in Scheme 76, which is similar to the previously reported transformations after generation of trifluoromethyl radical. TFSP was reduced by the excited photocatalyst, Ir(ppy)_3_* via a single-electron transfer affording the radical intermediate **102**. Then, homolysis of intermediate **102** gives CF_3_SO_2_ radical, which further gives trifluoromethyl radical after release of SO_2_. After generation of trifluoromethyl radical, the reaction proceeds through similar steps to form the final product **92**.

Asymmetric radical difunctionalization of alkenes remains a great challenge due to the short life of radicals and the extreme difficulty of stereochemical control of reactive radical intermediate. On the other hand, asymmetric radical reactions catalyzed by the combination of Cu salt/chiral ligand have been developed as an efficient strategy for the synthesis of varieties of chiral trifluoromethyl-containing compounds, as those reactions usually provide high stereoselectivity due to the formation of chiral Cu(III) complex intermediate.

**Scheme 77:**
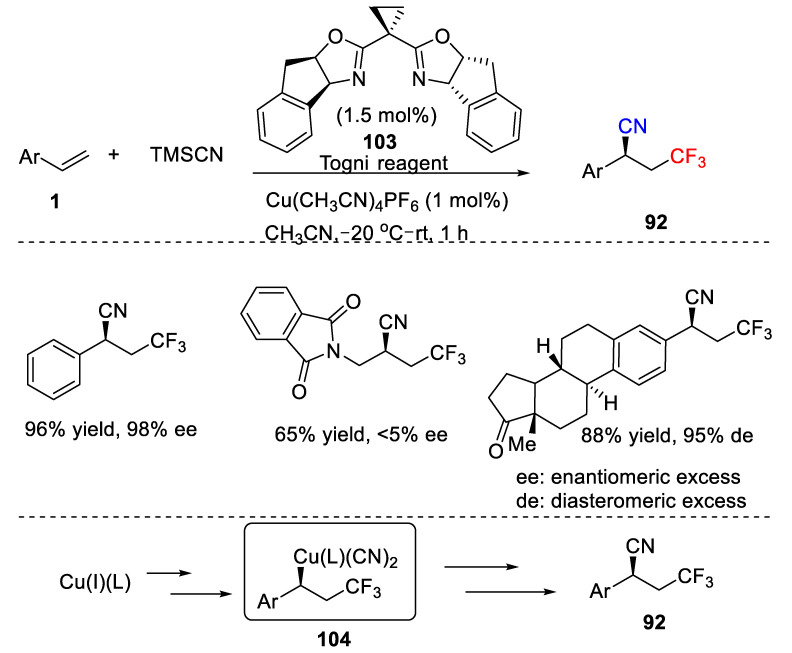
Asymmetric Cu-catalyzed cyanotrifluoromethylation reaction.

The pioneering work on enantioselective radical cyanotrifluoromethylation of alkenes was developed by Liu and coworkers in 2016 (Scheme 77) [108]. This asymmetric radical difunctionalization reaction used Cu(CH_3_CN)_4_PF_6_ as catalyst and 3-bis-2-oxazoline derivate ((3a*S*,3a’*S*,8a*R*,8a’*R*)-2,2’-(cyclopropane-1,1-diyl)bis(8,8a-dihydro-3aH-indeno[1 ,2-d]oxazole)) **103** as chiral ligand, affording trifluoromethylated alkylnitriles **92** in excellent chemical yields and high enantioselectivities. Interestingly, this reaction features a quite broad substrate scope which was generally not sensitive to the substitution pattern. It should be mentioned that the reaction only tolerated aryl substituted alkenes with good reaction outcomes; however, the terminal alkenes with an alkyl substituent could still provide the corresponding product but with poor enantioselectivities.

As indicated by the authors, this Cu-catalyzed reaction was also triggered by a SET to generate CF_3_ radical, followed by an addition to styrene to generate a benzylic radical intermediate. Then, the CF_3_ radical was trapped by Cu(II) to form a key chiral Cu(III) intermediate **104**, which proceeds through a reductive elimination to give the desired product.

Notably, the Han group also reported independently a similar catalytic enantioselective cyano-trifluoromethylation of styrenes with CuF_2_/chiral bidentate bis(oxazoline) ligand as catalyst to give trifluoromethyl nitriles in good yields and excellent enantioselectivities [109].

**Scheme 78:**
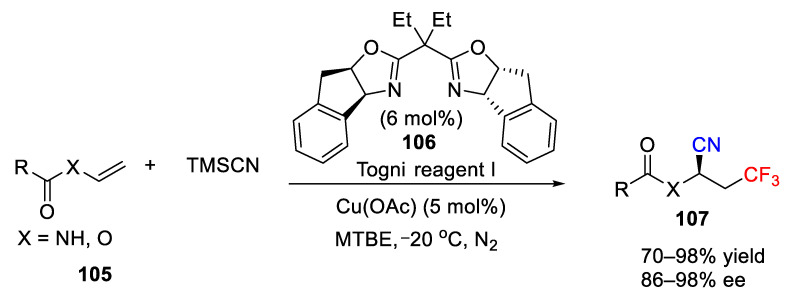
Asymmetric Cu-catalyzed cyanotrifluoromethylation reaction of enamides and vinyl esters.

Afterward, the same group demonstrated a related asymmetric catalytic system with Cu(OAc) and chiral bis-oxazoline ligand **106** as catalyst in 2020 to access a series of chiral α-cyano amides, α-cyano esters by use of enamides, and vinyl esters **105** as starting alkene substrates (Scheme 78) [110]. The key success of this reaction is the use of alkenes adjacent to a nitrogen atom or an oxygen atom, and the reaction proceeds via a key asymmetric coupling step of carbon-centered radical adjacent to heteroatom, affording the corresponding products **107** in good to excellent yields (70–98%) and enantioselectivities up to 98%.

Considering the advantages of the Cu/chiral ligand-catalyzed radical rely on strategy, then, the same group extended this asymmetric system from enamides to alkyl-substituted terminal alkenes featuring carbonyl unit, including ketone, amide, and ester in 2020 [111]. The reaction showed a wide substrate scope and a variety of substituents was tolerated to afford the corresponding product in excellent yields, however, with slightly lower stereoselectivities.

Due to the advantage of introducing two groups at the same time (namely, cyano and trifluoromethyl), as well as the good functional group tolerance, and mild reaction conditions, the cyanotrifluoromethylation difunctionalization strategy also gained wide recognition in other chemical platforms, such as remote cyanotrifluoromethyl difunctionalization [112,113,114] and construction of functionalized cyclic compounds [115,116,117].

## 6. Conclusions

As follows from the discussed data, the area of halo-trifluoromethylation of unsaturated bonds is a flourishing field of research. These reactions allow for a rapid access to CF_3_-containing building blocks which can be further elaborated to even more complex compounds using the halogen atom in vicinal position the CF_3_ group. Similar synthetic bonus a provided by cyano-trifluoromethylation approach where the CN function can be further transformed to a carboxylic group, or its derivative. One of the remarkable observations is the exceptional substrate generality of these reactions allowing additions to alkenes, alkynes and enynes. Also deserving special comment is a wide variety of reactions conditions that can be used to perform these transformations. As a note of critique, one can mention rather an insufficient number of stereoselective transformations, in particular, enantioselective formation of the CF_3_-C bond. Considering the key importance of stereogenic centers in the development of pharmaceutical drugs, we can expect some progress in this regard in the future. In general, the results reported so far bode quite well for this field to take a central stage in synthetic fluorine chemistry focused on the innovative development of fluorine-containing biologically active compounds and materials. 

## References

[1] Wang J., Sánchez-Roselló M., Aceña J.L., del Pozo C., Sorochinsky A.E., Fustero S., Soloshonok V.A., Liu H. (2013). Fluorine in Pharmaceutical Industry: Fluorine-Containing Drugs Introduced to the Market in the Last Decade (2001–2011). Chem. Rev..

[2] Zhou Y., Wang J., Gu Z., Wang S., Zhu W., Acena J.L., Soloshonok V.A., Izawa K., Hong L. (2016). Next Generation of Fluo-rine-Containing Pharmaceuticals, Compounds Currently in Phase II–III Clinical Trials of Major Pharmaceutical Companies: New Structural Trends and Therapeutic Areas. Chem. Rev..

[3] Mei H., Han J., Fustero S., Medio-Simon M., Sedgwick D.M., Santi C., Ruzziconi R., Soloshonok V.A. (2019). Fluorine-Containing Drugs Approved by the FDA in 2018. Chem. - A Eur. J..

[4] Zhu W., Wang J., Wang S., Gu Z., Aceña J.L., Izawa K., Liu H., Soloshonok V.A. (2014). Recent advances in the trifluoromethylation methodology and new CF3-containing drugs. J. Fluor. Chem..

[5] Mei H., Remete A.M., Zou Y., Moriwaki H., Fustero S., Lorand K., Soloshonok V.A., Han J. (2020). Fluorine-containing drugs approved by the FDA in 2019. Chin. Chem. Lett..

[6] Okuso S., Kawai H., Yasuda Y., Sugita Y., Kitayama T., Tokunaga E., Shibata N. (2014). Asymmetric Synthesis of Efavirenz via Or-ganocatalyzed Enantioselective Trifluoromethylation. Asian J. Org. Chem..

[7] Aceña J.L., Sorochinsky A.E., Soloshonok V.A. (2012). Recent advances in asymmetric synthesis of α-(trifluoromethyl)-containing α-amino acids. Synthesis..

[8] Turcheniuk K.V., Kukhar V.P., Röschenthaler G.-V., Aceña J.L., Soloshonok V.A., Sorochinsky A.E. (2013). Recent advances in the synthesis of fluorinated aminophosphonates and aminophosphonic acids. RSC Adv..

[9] Yu Y., Liu A., Dhawan G., Mei H., Zhang W., Izawa K., Soloshonok V.A., Han J. (2021). Fluorine-containing pharmaceuticals approved by the FDA in 2020: Synthesis and biological activity. Chin. Chem. Lett..

[10] Han J., Kiss L., Mei H., Remete A.M., Ponikvar-Svet M., Sedgwick D.M., Roman R., Fustero S., Moriwaki H., Soloshonok V.A. (2021). Chemical Aspects of Human and Environmental Overload with Fluorine. Chem. Rev..

[11] Han J., Remete A.M., Dobson L.S., Kiss L., Izawa K., Moriwaki H., Soloshonok V.A., O’Hagan D. (2020). Next generation orga-nofluorine containing blockbuster drugs. J. Fluor. Chem..

[12] Mei H., Han J., White S., Graham D.J., Izawa K., Sato T., Fustero S., Meanwell N.A., Soloshonok V.A. (2020). Tailor-Made Amino Acids and Fluorinated Motifs as Prominent Traits in Modern Pharmaceuticals. Chem. - A Eur. J..

[13] Mei H., Han J., Klika K.D., Izawa K., Sato T., Meanwell N.A., Soloshonok V.A. (2019). Applications of fluorine-containing amino acids for drug design. Eur. J. Med. Chem..

[14] Gillis E.P., Eastman K.J., Hill M.D., Donnelly D., Meanwell N. (2015). Applications of Fluorine in Medicinal Chemistry. J. Med. Chem..

[15] Inoue M., Sumii Y., Shibata N. (2020). Contribution of Organofluorine Compounds to Pharmaceuticals. ACS Omega..

[16] Ogawa Y., Tokunaga E., Kobayashi O., Hirai K., Shibata N. (2020). Current contributions of organofluorine compounds to the agro-chemical industry. Iscience.

[17] Johnson B.M., Shu Y.-Z., Zhuo X., Meanwell N.A. (2020). Metabolic and Pharmaceutical Aspects of Fluorinated Compounds. J. Med. Chem..

[18] Yang X., Wu T., Phipps R.J., Toste F.D. (2014). Advances in Catalytic Enantioselective Fluorination, Mono-, Di-, and Trifluoromethylation, and Trifluoromethylthiolation Reactions. Chem. Rev..

[19] Egami H., Sodeoka M. (2014). Trifluoromethylation of Alkenes with Concomitant Introduction of Additional Functional Groups. Angew. Chem. Int. Ed..

[20] Pan J., Wu J., Wu F. (2021). Progress in Fluoroalkylation of Multicomponent. Chin. J. Org. Chem..

[21] Qiu Y., Wei F., Ye L., Zhao M. (2021). Advances in Trifluorometylation-Promoted Functional Group Migration of Alkenes. Chin. J. Org. Chem..

[22] Studer A. (2012). A “renaissance” in radical trifluoromethylation. Angew. Chem. Int. Ed..

[23] Koike T., Akita M. (2014). Trifluoromethylation by Visible-Light-Driven Photoredox Catalysis. Top. Catal..

[24] Barata-Vallejo S., Lantaño B., Postigo A. (2014). Recent Advances in Trifluoromethylation Reactions with Electrophilic Trifluoromethylating Reagents. Chem. - A Eur. J..

[25] Umemoto T. (1996). Electrophilic Perfluoroalkylating Agents. Chem. Rev..

[26] Charpentier J., Früh N., Togni A. (2014). Electrophilic Trifluoromethylation by Use of Hypervalent Iodine Reagents. Chem. Rev..

[27] Prier C.K., Rankic D.A., Macmillan D.W.C. (2013). Visible Light Photoredox Catalysis with Transition Metal Complexes: Applications in Organic Synthesis. Chem. Rev..

[28] Alonso C., de Marigorta E.M., Rubiales G., Palacios F. (2015). Carbon Trifluoromethylation Reactions of Hydrocarbon Derivatives and Heteroarenes. Chem. Rev..

[29] Ouyang Y., Xu X.-H., Qing F.-L. (2018). Trifluoromethanesulfonic Anhydride as a Low-Cost and Versatile Trifluoromethylation Reagent. Angew. Chem. Int. Ed..

[30] Chen S., Feng D.-F., Li D.-Y., Liu P.-N. (2018). Radical Cyanotrifluoromethylation of Isocyanides: Step-Economical Access to CF3-Containing Nitriles, Amines, and Imines. Org. Lett..

[31] Oh H., Park A., Jeong K.-S., Han S.B., Lee H. (2019). Copper-Catalyzed 1, 2-Bistrifluoromethylation of Terminal Alkenes. Adv. Synth. Catal..

[32] Oh S.H., Malpani Y.R., Ha N., Jung Y.-S., Han S.B. (2014). Vicinal Difunctionalization of Alkenes: Chlorotrifluoromethylation with CF3SO2Cl by Photoredox Catalysis. Org. Lett..

[33] Maeda K., Kurahashi T., Matsubara S. (2019). Chlorotrifluoromethylation of Terminal Olefins by Atom Transfer-Type Radical Reaction Catalyzed by Cobalt Complexes. Eur. J. Org. Chem..

[34] Zhang W., Lin J.-H., Xiao J.-C. (2018). Cu-catalyzed chlorotrifluoromethylation of alkenes with CF3SO2Cl. J. Fluor. Chem..

[35] Lin J.-S., Wang F.-L., Dong X.-Y., He W.-W., Yuan Y., Chen S., Liu X.-Y. (2017). Catalytic asymmetric radical aminoperfluoroalkylation and aminodifluoromethylation of alkenes to versatile enantioenriched-fluoroalkyl amines. Nat. Commun..

[36] Tang X.-J., Dolbier W.R. (2015). Efficient Cu-catalyzed atom transfer radical addition reactions of fluoroalkylsulfonyl chlorides with electron-deficient alkenes induced by visible light. Angew. Chem. Int. Ed..

[37] Tang X.-J., Thomoson C.S., Dolbier W.R. (2014). Photoredox-Catalyzed Tandem Radical Cyclization of N-Arylacrylamides: General Methods To Construct Fluorinated 3,3-Disubstituted 2-Oxindoles Using Fluoroalkylsulfonyl Chlorides. Org. Lett..

[38] Bagal D.B., Kachkovskyi G., Knorn M., Rawner T., Bhanage B.M., Reiser O. (2015). Trifluoromethylchlorosulfonylation of Alkenes: Evidence for an Inner-Sphere Mechanism by a Copper Phenanthroline Photoredox Catalyst. Angew. Chem. Int. Ed..

[39] Murat Alkan-Zambada M., Hu X. (2018). Cu Photoredox Catalysts Supported by a 4, 6-Disubstituted 2, 2′-Bipyridine Ligand: Application in Chlorotrifluoromethylation of Alkenes. Organometallics..

[40] Lee K., Lee S., Kim N., Kim S., Hong S. (2020). Visible-Light-Enabled Trifluoromethylative Pyridylation of Alkenes from Pyridines and Triflic Anhydride. Angew. Chem. Int. Ed..

[41] Ouyang Y., Tong C.-L., Xu X.-H., Qing F.-L. (2020). Copper and Zinc Copromoted Bromo(chloro)trifluoromethylation of Alkenes and Alkynes with Trifluoromethanesulfonic Anhydride. Org. Lett..

[42] Han H.S., Lee Y.J., Jung Y.-S., Han S.B. (2017). Stereoselective photoredox-catalyzed chlorotrifluoromethylation of alkynes: Synthesis of tetrasubstituted alkenes. Org. Lett..

[43] Ma J.-J., Yi W.-B., Lu G.-P., Cai C. (2015). Decarboxylative and Denitrative Trifluoromethylation for the Synthesis of Cvinyl-CF3Compounds with Togni (II) Reagent. Adv. Synth. Catal..

[44] Thomosom C.S., Tang X.-J., Dolbier W.R. (2015). Chloro, Difluoromethylation and Chloro, Carbomethoxydifluoromethylation: Reaction of Radicals Derived from RfSO2Cl with Unactivated Alkenes under Metal-Free Conditions. J. Org. Chem..

[45] O’Hara F., Blackmond D.G., Baran P.S. (2013). Radical-based regioselective C-H functionalization of electron-deficient heteroarenes: Scope, tunability, and predictability. J. Am. Chem. Soc..

[46] Otsuka T., Ishii A., Dub P.A., Ikariya T. (2013). Practical Selective Hydrogenation of α-Fluorinated Esters with Bifunctional Pincer-Type Ruthenium(II) Catalysts Leading to Fluorinated Alcohols or Fluoral Hemiacetals. J. Am. Chem. Soc..

[47] Yang B., Xu X.-H., Qing F.-L. (2015). Iron-Mediated Chlorotrifluoromethylation of Alkenes with Sodium Trifluoromethanesulfinate. Chin. J. Chem..

[48] Zhang C. (2014). Application of Langlois’ Reagent in Trifluoromethylation Reactions. Adv. Synth. Catal..

[49] Fang J., Wang Z.K., Wu S.W., Shen W.G., Ao G.Z., Liu F. (2017). Photoredox-catalysed chloro-, bromo-and trifluoromethyl-thio-trifluoromethylation of unactivated alkenes with sodium triflinate. Chem. Commun..

[50] Hou H., Tang D., Li H., Xu Y., Yan C., Shi Y., Chen X., Zhu S. (2019). Visible-Light-Driven Chlorotrifluoromethylative and Chloro-trichloromethylative Cyclizations of Enynes. J. Org. Chem..

[51] Hou H., Li H., Xu Y., Song C., Wang C., Shi Y., Hang Y., Yang C., Zhu S. (2018). Visible-Light-Mediated Chlorosulfonylative Cycli-zations of 1, 6-Enynes. Adv. Synth. Catal..

[52] Ye K.-Y., Song Z., Sauer G.S., Harenberg J.H., Fu N., Lin S. (2018). Synthesis of Chlorotrifluoromethylated Pyrrolidines by Electrocatalytic Radical Ene-Yne Cyclization. Chem. – A Eur. J..

[53] Ye K.-Y., Pombar G., Fu N., Sauer G.S., Keresztes I., Lin S. (2018). Anodically Coupled Electrolysis for the Heterodifunctionalization of Alkenes. J. Am. Chem. Soc..

[54] Guo J.-Y., Wu R.-X., Jin J.-K., Tian S.-K. (2016). TfNHNHBoc as a Trifluoromethylating Agent for Vicinal Difunctionalization of Terminal Alkenes. Org. Lett..

[55] Xu C., Song X., Guo J., Chen S., Gao J., Jiang J., Gao F., Li Y., Wang M. (2018). Synthesis of Chloro(phenyl)trifluoromethyliodane and Catalyst-Free Electrophilic Trifluoromethylations. Org. Lett..

[56] Xu C., Huang W., Zhang R., Gao C., Li Y., Wang M. (2019). Trifluoromethylations of Alkenes Using PhICF3Cl as Bifunctional Reagent. J. Org. Chem..

[57] Fu M., Chen L., Jiang Y., Jiang Z.-X., Yang Z. (2016). Copper-Catalyzed Intermolecular Chloro- and Bromotrifluoromethylation of Alkenes. Org. Lett..

[58] Masson G., Carboni A., Dagousset G., Magnier E. (2015). Three-Component Photoredox-Mediated Chloro-, Bromo-, or Iodotrifluoromethylation of Alkenes. Synthesis..

[59] Hang Z., Li Z., Liu Z.-Q. (2014). Iodotrifluoromethylation of Alkenes and Alkynes with Sodium Trifluoromethanesulfinate and Iodine Pentoxide. Org. Lett..

[60] Liu Z.-Q., Liu D. (2017). Free-Radical Bromotrifluoromethylation of Olefin via Single-Electron Oxidation of NaSO2CF3 by NaBrO3. J. Org. Chem..

[61] Shang X.J., Liu D., Liu Z.Q. (2018). A NaSO2CF3/NaBrO3-mediated bromotrifluoromethylation of enyne via free-radical cascade processes. Org. Chem. Front..

[62] Huang L., Zheng S.-C., Tan B., Liu X.-Y. (2015). Trifluoromethylation-Initiated Remote Cross-Coupling of Carbonyl Compounds to Form Carbon-Heteroatom/Carbon Bonds. Chem. - A Eur. J..

[63] Wang X., Lei J., Liu Y., Ye Y., Li J., Sun K. (2021). Fluorination and fluoroalkylation of alkenes/alkynes to construct fluoro-containing heterocycles. Org. Chem. Front..

[64] Shaw M.H., Twilton J., MacMillan D.W.C. (2016). Photoredox Catalysis in Organic Chemistry. J. Org. Chem..

[65] Hockin B.M., Li C., Robertson N., Zysman-Colman E. (2019). Photoredox catalysts based on earth-abundant metal complexes. Catal. Sci. Technol..

[66] Iqbal N., Jung J., Park S., Cho E.J. (2013). Controlled Trifluoromethylation Reactions of Alkynes through Visible-Light Photoredox Catalysis. Angew. Chem. Int. Ed..

[67] Ravelli D., Fagnoni M. (2011). Dyes as Visible Light Photoredox Organocatalysts. ChemCatChem.

[68] Wei G., Basheer C., Tan C.-H., Jiang Z. (2016). Visible light photocatalysis in chemoselective functionalization of C(sp 3) H bonds enabled by organic dyes. Tetrahedron Lett..

[69] Koike T. (2020). Frontiers in Radical Fluoromethylation by Visible-Light Organic Photocatalysis. Asian J. Org. Chem..

[70] Romero N., Nicewicz D.A. (2016). Organic Photoredox Catalysis. Chem. Rev..

[71] Park G.-R., Choi Y., Choi M.G., Chang S.K., Cho E.J. (2017). Metal-Free Visible-Light-Induced Trifluoromethylation Reactions. Asian J. Org. Chem..

[72] Beniazza R., Atkinson R., Absalon C., Castet F., Denisov S.A., McClenaghan N.D., Lastécouères D., Vincent J.-M. (2016). Benzophenonevs. Copper/Benzophenone in Light-Promoted Atom Transfer Radical Additions (ATRAs): Highly Effective Iodoperfluoroalkylation of Alkenes/Alkynes and Mechanistic Studies. Adv. Synth. Catal..

[73] Beniazza R., Douarre M., Lastécouères D., Vincent J.-M. (2017). Metal-free and light-promoted radical iodotrifluoromethylation of alkenes with Togni reagent as the source of CF3 and iodine. Chem. Commun..

[74] Beniazza R., Remisse L., Jardel D., Lastécouères D., Vincent J.-M. (2018). Light-mediated iodoperfluoroalkylation of alkenes/alkynes catalyzed by chloride ions: Role of halogen bonding. Chem. Commun..

[75] Jentzsch A.V., Emery D., Mareda J., Nayak S.K., Metrangolo P., Resnati G., Sakai N., Matile S. (2012). Transmembrane anion transport mediated by halogen-bond donors. Nat. Commun..

[76] Yajima T., Murase M., Ofuji Y. (2020). Visible Light-Induced Radical Iodoperfluoroalkylation of Unactivated Olefins Cooperatively Cat-alyzed by Enamines and Amines. Eur. J. Org. Chem..

[77] Zeng Y., Zhang L., Zhao Y., Ni C., Zhao J., Hu J. (2013). Silver-Mediated Trifluoromethylation–Iodination of Arynes. J. Am. Chem. Soc..

[78] An W., Ha N., Lee H.M., Malpani Y.R., Lee D.H., Jung Y.S., Han S.B. (2015). Copper-Mediated Halotrifluoromethylation of Unacti-vated Alkenes. Adv. Synth. Catal..

[79] Xu T., Cheung C.W., Hu X. (2014). Iron-Catalyzed 1,2-Addition of Perfluoroalkyl Iodides to Alkynes and Alkenes. Angew. Chem. Int. Ed..

[80] Janson P.G., Ghoneim I., Ilchenko N.O., Szabó K.J. (2012). Electrophilic Trifluoromethylation by Copper-Catalyzed Addition of CF3-Transfer Reagents to Alkenes and Alkynes. Org. Lett..

[81] Ilchenko N.O., Janson P.G., Szabo K.J. (2013). Copper-Mediated Cyanotrifluoromethylation of Styrenes Using the Togni Reagent. J. Org. Chem..

[82] Egami H., Usui Y., Kawamura S., Nagashima S., Sodeoka M. (2015). Product control in alkene trifluoromethylation: Hydrotrifluoro-methylation, vinylic trifluoromethylation, and iodotrifluoromethylation using Togni Reagent. Chem. Asian J..

[83] Dye J.L. (1990). Electrides: Ionic Salts with Electrons as the Anions. Science.

[84] Choi S., Kim Y.J., Kim S.M., Yang J.W., Kim S.W., Cho E.J. (2014). Hydrotrifluoromethylation and iodotrifluoromethylation of alkenes and alkynes using an inorganic electride as a radical generator. Nat. Commun..

[85] Rosso C., Williams J.D., Filippini G., Prato M., Kappe C.O. (2019). Visible-Light-Mediated Iodoperfluoroalkylation of Alkenes in Flowand Its Application to the Synthesis of a Key FulvestrantIntermediate. Org. Lett..

[86] Rawner T., Lutsker E., Kaiser C.A., Reiser O. (2018). The Different Faces of Photoredox Catalysts: Visible-Light-MediatedAtom Transfer Radical Addition (ATRA) Reactions of PerfluoroalkylIodides with Styrenes and Phenylacetylenes. ACS Catal..

[87] Mao T., Ma M.J., Zhao L., Xue D.P., Yu Y., Gu J., He C.Y. (2020). A general and green fluoroalkylation reactionpromotedvianoncovalent interactions betweenacetone and fluoroalkyl iodides. Chem. Commun..

[88] Du Y., Pearson R.M., Lim C.H., Sartor S.M., Ryan M.D., Yang H., Damrauer N.H., Miyake G.M. (2017). Strongly Reducing, Visible-LightOrganic Photoredox Catalysts asSustainable AlternativestoPrecious Metals. Chem. Eur. J..

[89] Kawamura S., Sekine D., Sodeoka M. (2017). Synthesis of CF3-containing oxazolines via trifluoromethylation ofallylamides with Togni reagent in the presence of alkali metal iodides. J. Fluor. Chem..

[90] Sun H., Cui G., Shang H., Cui B. (2020). Mn(OAc)3-Mediated Addition Reactions of NaSO2CF3andPerhalogenated Carboxylic Acids with Unactivated AlkenesConjectured by a Single Electron Transfer and Halogen AbstractionMechanism. J. Org. Chem..

[91] Kostromitin V.S., Zemtsov A.A., Kororekin V.A., Levin V.V., Dilman A.D. (2021). Atom-transfer radical addition of fluoroalkylbromides to alkenesviaa photoredox/coppercatalytic system. Chem. Commun..

[92] Ol’shevskaya V.A., Tyutyunov A.A., Ibragimova L.F., Kononova E.G., Rys E.G. (2019). Facile synthetic route to fluoroalkylated car-boranes by copper-catalyzedreaction of fluoroalkane sulfonyl bromides with allyl carboranes. Polyhedron..

[93] Quan Y., Shi W., Song Y., Jiang X., Wang C., Lin W. (2021). Bifunctional Metal−Organic Layer with Organic Dyes and IronCenters for Synergistic Photoredox Catalysis. J. Am. Chem. Soc..

[94] Eng S., Reiser O. (2020). Making Copper Photocatalysis Even More Robust and Economic: Photoredox Catalysis with [CuII(dmp)2Cl]Cl. Eur. J. Org. Chem..

[95] Huang W., Xu C., Yu J., Wang M. (2021). ZnI2-Catalyzed Aminotrifluoromethylation Cyclization of AlkenesUsing PhICF3Cl. J. Org. Chem..

[96] Yu W., Xu X.-H., Qing F.-L. (2015). Silver-Mediated Oxidative Fluorotrifluoromethylation of Unactivated Alkenes. Adv. Synth. Catal..

[97] Liu Z., Chen H., Lv Y., Tan X., Shen H., Yu H.-Z., Li C. (2018). Radical Carbofluorination of Unactivated Alkenes with Fluoride Ions. J. Am. Chem. Soc..

[98] Sato A., Ponomarenko M.V., Ono T., Röschenthaler G.V., Soloshonok V.A. (2019). Mediator and Additive Free Trifluoromethyl-Fluorination of Terminal Alkenes by Persistent Perfluoroalkyl Radical. Eur. J. Org. Chem..

[99] Zhang S.-L., Wan H.-X., Bie W.-F. (2017). syn-Fluoro- and -Oxy-trifluoromethylation of Arylacetylenes. Org. Lett..

[100] Zhang S.L., Dong J.J. (2019). Hydrogen-Bonding-Assisted α-F Elimination from Cu–CF3 for in Situ Generation of R3N·HF Reagents: Re-action Design and Applications. Org. Lett..

[101] Lee W., Lee Y., Yoo M., Han S.B., Kim H.J. (2020). Photoredox-catalyzed halotrifluoromethylations of alkynes with triethylammonium halides: Synthesis of tetrasubstituted alkenes containing CF3 and halogens. Org. Chem. Front..

[102] He Y.T., Li L.H., Yang Y.F., Zhou Z.Z., Hua H.L., Liu X.Y., Liang Y.M. (2014). Copper-Catalyzed Intermolecular Cyanotrifluoro-methylation of Alkenes. Org. Lett..

[103] He Y.-T., Wang Q., Zhao J., Liu X.-Y., Xu P.-F., Liang Y.-M. (2015). The copper-catalyzed synthesis of β-trifluoromethylated acrylonitriles and trifluoromethyl-substituted 2H-azirines. Chem. Commun..

[104] Liang Z., Wang F., Chen P., Liu G. (2014). Copper-catalyzed intermolecular cyanotrifluoromethylation of alkenes: Convenient synthesis of CF3-containing alkyl nitriles. J. Fluor. Chem..

[105] Guo Q., Wang M., Wang Y., Xu Z., Wang R. (2017). Photoinduced, copper-catalyzed three components cyanofluoroalkylation of alkenes with fluoroalkyl iodides as fluoroalkylation reagents. Chem. Commun..

[106] Zhu Y., Tian J., Gu X., Wang Y. (2018). Free-Radical-Promoted Copper-Catalyzed Intermolecular Cyanosulfonylation and Cyanotri-fluoromethylation of Unactivated Alkenes in Water-Containing Solvents. J. Org. Chem..

[107] Zhang M., Lin J.-H., Xiao J.-C. (2021). A Readily Available Trifluoromethylation Reagent and Its Difunctionalization of Alkenes. Org. Lett..

[108] Wang F., Wang D., Wan X., Wu L., Chen P., Liu G. (2016). Enantioselective Copper-Catalyzed Intermolecular Cyanotrifluoromethyla-tion of Alkenes via Radical Process. J. Am. Chem. Soc..

[109] Sha W., Zhu Y., Mei H., Han J., Soloshonok V.A., Pan Y. (2017). Catalytic Enantioselective Cyano-Trifluoromethylation of Styrenes. Chem. Select..

[110] Zhang G., Zhou S., Fu L., Chen P., Li Y., Zou J., Liu G. (2020). Asymmetric Coupling of Carbon-Centered Radicals Adjacent to Nitrogen: Copper-Catalyzed Cyanation and Etherification of Enamides. Angew. Chem. Int. Ed..

[111] Zhou S., Zhang G., Fu L., Chen P., Li Y., Liu G. (2020). Copper-Catalyzed Asymmetric Cyanation of Alkenes via Carbonyl-Assisted Coupling of Alkyl-Substituted Carbon-Centered Radicals. Org. Lett..

[112] Zhang Z.Q., Meng X.Y., Sheng J., Lan Q., Wang X.S. (2019). Enantioselective Copper-Catalyzed 1,5-Cyanotrifluoromethylation of Vi-nylcyclopropanes. Org. Lett..

[113] He Y.T., Li L.H., Zhou Z.Z., Hua H.L., Qiu Y.F., Liu X.Y., Liang Y.M. (2014). Copper-Catalyzed Three-Component Cyanotrifluoro-methylation/Azidotrifluoromethylation and Carbocyclization of 1,6-Enynes. Org. Lett..

[114] Wang F., Wang D., Zhou Y., Liang L., Lu R., Chen P., Lin Z., Liu G. (2018). Divergent Synthesis of CF3-Substituted Allenyl Nitriles by Ligand-Controlled Radical 1,2- and 1,4-Addition to 1,3-Enynes. Angew. Chem. Int. Ed..

[115] Zou H.D., Cui C.C., Guo C., Hao W.J., Tu S.J., Jiang B. (2020). Metal-Catalyzed Spiroannulation-Fluoromethylfunctionaliztions of 1,5-Enynes for the Synthesis of Stereodefined (Z)-Spiroindenes. Chem. Asian J..

[116] Zhang T.S., Hao W.J., Cai P.J., Li G., Tu S.J., Jiang B. (2020). Copper-Catalyzed Annulation–Cyanotrifluoromethylation of 1,6-Enynes Toward 1-Indanones via a Radical Process. Front. Chem..

[117] Ji W.-Z., Shi H.-N., Hao W.-J., Wei P., Tu S.-J., Jiang B. (2020). Generation of stereodefined (Z)-3,4-dihydronaphthalen-1(2H)-ones via copper-catalyzed annulation-cyanotrifluoromethylation of 1,7-enynes. Tetrahedron Lett..

